# Acknowledgement to Reviewers of *International Journal of Molecular Sciences* in 2015

**DOI:** 10.3390/ijms17010142

**Published:** 2016-01-21

**Authors:** International Journal of Molecular Sciences Editorial Office

**Affiliations:** MDPI AG, Klybeckstrasse 64, CH-4057 Basel, Switzerland; ijms@mdpi.com

The editors of *International Journal of Molecular Sciences* would like to express their sincere gratitude to the following reviewers for assessing manuscripts in 2015.

We greatly appreciate the contribution of expert reviewers, which is crucial to the journal’s editorial decision-making process. Several steps have been taken in 2015 to thank and acknowledge reviewers. Good, timely reviews are rewarded with a discount off their next MDPI publication. By creating an account on the submission system, reviewers can access details of their past reviews, see the comments of other reviewers, and download a letter of acknowledgement for their records. In addition, MDPI has launched a collaboration with Publons, a website that seeks to publicly acknowledge reviewers on a per journal basis. This is all done, of course, within the constraints of reviewer confidentiality. Feedback from reviewers shows that most see their task as a voluntary and mostly unseen work in service to the scientific community. We are grateful to our reviewers for the contribution they make.

Abbiati, GiorgioHoffman, HeinrichPelkonen, OlaviAbbott, Karen L.Hoffman, Steven A.Pellegrini-Giampietro, D.E.Abbt-Braun, GudrunHoffmann, KlausPelletier, StephaneAbdallah, Chadi G.Hoffmann, Markus H.Pelosi, PaoloAbdel-Mottaleb, M.M.A.Hoffmann, MathiasPena, Romi N.Abenavoli, LudovicoHöfinger, SiegfriedPenders, Thomas M.Abend, MichaelHofmann, JohannPenfold, Scott N.Abou Neel, Ensanya A.Hogan, AndrewPenfornis, PatriceAbraham, SolanaHohenegger, MartinPeng, Wen-HuangAbusco, Anna ScottoHojjat-Farsangi, M.Peng, ZhenlingAcién Fernández, F.G.Holden, PatriciaPennypacker, KeithAcord, JohnHolding, David R.Penzes, PeterAcuña, DaríoHolen, IngunnPepys, MarkAdamakis, I.-D.S.Holick, Michael F.Peral, BelénAdams, James B.Holland, Peter W.H.Perales, OscarAdler, CarrieHollander, W. denPerego, CarlaAdriano, MaroccoHollenstein, MarcelPereira, C.S.A.Afantitis, AntreasHolliday, DawnPereira, DavidAfolayan, A.J.Holman, TheodorePereira, FlorbelaAgostini, MarcoHolmberg, Scott D.Pereira, LeonelAgostino, MarkHolmes, RobertPereira, Olívia R.Agoston, Denes V.Holmstrøm, KimPerepelyuk, MarynaAharoni, RinaHolst, Jens JuulPeretz, AviAhlenstiel, GoloHöltinger, StefanPérez, Emilio M.Ahmad, Faiz U.Hondan, TomoyukiPérez, MontseAhmed, HafizHong, Mee YoungPerez-Cano, FranciscoAhn, Sang-hoonHong, YonggeunPérez-Castrillón, José L.Aikawa, ShimpeiHonoré, Patrick M.Perez-Jimenez, RaulAikawa, TakuyaHool, Livia C.Pérez-Sánchez, JaumeAinscough, Justin F.X.Hordvik, IvarPérigo, E.A.Akazawa, ChihiroHorii, TakuroPerks, Claire M.Akbar, S.M.F.Horn, R.Perl, AndrasAkbari, MansourHorovitz, AmnonPerona, John J.Akbari, Mohammad R.Hörsch, DieterPerret, XavierAkbarzadeh, A.H.Hortelano, SonsolesPerricone, CarloAkeda, KojiHorvat, JosipPerrier, AurélieAkita, SadanoriHorvath, David P.Perrine-Walker, F.M.Akopian, Armen N.Hossein-Babaei, FarazPerron, KarlAl Ghouleh, ImadHou, Chien-WeiPersoz, CharlesAlabugin, IgorHou, JianghuiPersson, KennethAl-Ahmadie, Hikmat A.Hou, SusanPersson, RasmusAlavez, SilvestreHoude, MarioPerumal, DeepakAlavi, SamanHouee-Levin, ChantalPeruzzi, FrancescaAlbalat, AmayaHoung, Jer-yiingPeterman, Erwin J.G.Albero, CenciHoury, Walid A.Peters, ReneAlcoutlabi, MatazHoushmand, MassoudPetersen, Christian P.Al-Ejeh, FaresHouston, Kevin D.Petersen, ElijahAleman, SooHouston, Mark C.Peterson, JuliaAlessandro, PasseraHouston, RossPetrlova, JitkaAlevizos, IliasHowell, TysonPezzotti, GiuseppeAlexeyev, MikhailHowell, ViivePflugmacher, StephanAlexiou, ChristophHoyer-Fender, SigridPhillips, Jacqueline K.Alexis, FrankHsia, Shih-MinPhilpott, MikeAlfaidy, N.Hsieh, Chung-BaoPi, LiyaAlfandari, DominiqueHsieh, Yi-HsienPiazza, FabrizioAlfonso, IgnacioHsu, Chin-linPiccialli, GennaroAlfonso, MiguelHsu, Chung Y.Piccinini, AnnaAlfonzo, AntonioHsu, Fu-yinPiccoli, G.B.Alikhani, EsmailHsu, Hsue-YinPichat, PierreAlison, Malcolm R.Hsu, TcPichler, MartinAlkan, NoamHsueh, Yi-HuangPickkers, PeterAl-Kateb, HussamHu, BoPicklo, Matthew J.Allahverdiyeva, YagutHu, MingKuanPiga, MatteoAllain, Jean-PierreHu, Valerie W.Piggins, Hugh D.Allinson, Sarah L.Hu, ZhuoweiPignata, ClaudioAlmasio, P.L.Huang, Chiu-jungPihlanto, AnneAlonso, Maria CarmenHuang, Chi-YuPillay, VinessAloulou, AhmedHuang, Chun-JungPillon, YohanAlpini, GianfrancoHuang, FeiPilmé, JulienAltarawneh, M.Huang, FeihePilon, MarcAltenbach, Susan B.Huang, Hsuan-ChengPilon-Thomas, ShariAlvarez-Suarez, J.M.Huang, JiweiPinalli, RobertaAly, HamedHuang, Li-FenPincus, Seth H.Alzaid, F.Huang, PengPino-Figueroa, AlejandroAmado, FranciscoHuang, Shih-mingPinton, PaoloAmarasinghe, V.Huang, ShuangPippucci, TommasoAmarenco, GerardHuang, Tim Hui-MingPires, J.C.M.Amatngalim, Gimano D.Huang, Wen-LiPirillo, AngelaAmbs, StefanHuang, Ya-lingPirola, CarlosAmi, DilettaHuang, Yi-WenPitchiaya, S.Amira, ZarroukHuang, YuPittelkow, MichaelAmmendola, RosarioHuber, VeronicaPittman, Jon K.Amodio, NicolaHuchzermeyer, BernhardPitto, LetiziaAmorati, RiccardoHull, JoePiva, TerrenceAmorós, AsunciónHulten, EdwardPlatt, Jeffrey L.An, WonsukHumblot, VincentPlatts, JamesAnastasi, EmanuelaHunter, Christopher A.Plett, JonathanAnchan, RaymondHuppertz, BertholdPliego-Alfaro, FernandoAnders, Hans-joachimHur, YoonkangPlourde, GuyAndersen, M.-B.S.Hura, TomaszPoblaciones, Maria J.Andersen, OlufHurlé, JuanPoerschmann, JuergenAndersen, SigveHussain, M MahmoodPognonec, PhilippeAnderson, Anne J.Hussein, MalikPointillart, FabriceAnderson, KurtHust, MichaelPoirier, IsabelleAnderson, Peter D.Hvidsten, Torgeir R.Poldermans, DonAndersson, JanHwa, JohnPolerecky, LubosAndjelkovic, Anuska V.Hwang, Daniel H.Polesskaya, AnnaAndrade, PaulaHwang, DavidPolishchuk, RomanAndreadou, IoannaHwang, Kuo-jenPollastri, GianlucaAndreana, Peter R.Hwang, Ming-JingPollmann, KatrinAngeli, CelestinoHwang, Sun WookPoma, AlessandroAngelico, FrancescoHwang, Yoon JungPomar, FedericoAngelov, Doychin N.Hyeon, ChangbongPomero, FulvioAnglani, FrancaHynes, KimPoncet, ValérieAnifandis, Georgios M.Hyun, Kyung-YaePonchon, LucAnraku, MakotoIagaru, AndreiPonomarev, Eugene D.Antoniadi, IoannaIanni, FedericaPonsaerts, PeterAntonio, BaltazarIasevoli, FelicePontaño, Francisco PérezAoki, KazuhiroIbarra Molero, BeatrizPook, Mark A.Aoki, YoshinoriIbekwe, MarkPopa-Wagner, AurelAparicio, FredericIbrahim, A.G.-E.Poptani, HarishApone, FabioIbrahim, Salam A.Pörtner, RalfAppel, JensIchikawa, HiroshiPosada Estefan, Olga M.Appenroth, KlausIdnurm, AlexanderPossee, RobertApte, SwapnaIdo, AkioPotaczek, Daniel P.Araki, WataruIdoyaga, JulianaPouli, NicoleAravindan, NatarajanIentile, RiccardoPoulos, Thomas L.Arbona, VicentIglesias, Domingo J.Powell, J.T.Arbones-Mainar, JoseIglesias-Fernández, R.Powell, N.Archer, TrevorIguchi, ErikoPowers, RobertArdell, DavidIhara, EijiPradhan, SriharsaArden, CatherineIkari, YujiPrado, Rafael deArgon, YairIkawa, MasahitoPrasad, SadheoArgyriou, NotisIkeda, Masahiro.Prasanth, K.V.Ariani, AndreaIlan, EzgiPrashanth, Konda G.Ariga, KatsuhikoIlling, Robert-BenjaminPrat, MariaAriño, CristinaIm, Hee-JeongPreissner, Klaus T.Arinzeh, T.L.Imai, HirotakaPrentis, Peter J.Arita, MinetaroImaizumi, KazunoriProestos, CharalamposAriunbaatar, JavkhlanImataka, HiroakiProust, HélèneAriyama, KaoruImmel, FrançoisePrudovsky, IgorArkudas, AndreasImokawa, GenjiPrywes, RonArmero Ibáñez, EvaImumorin, Ikhide G.Pucker, Andrew D.Armstrong, Ehrin J.Indolfi, GiuseppePuddu, Paolo Emilioarnhold, juergenIndriolo, EmilyPuentes, FabiolaAroca, RicardoInfascelli, F.Puértolas, EduardoArtlip, TimothyInoue, KeijiPuglisi, EdoardoArugula, MaryInselman, AmyPuinean, Alin M.Aruoma, OkezieInsenser, MaríaPulford, KarenAsahi, MichioInui, MasafumiPuppo, FrancescoAsakura, TomikoInukai, YoshiakiPutz, Mihai V.Asano, KoichiroInuzuka, HiroyukiQi, QungangAsatryan, LianaInvernizzi, PietroQi, YipingAshfaq, M.K.Ioelovich, MichaelQian, HaiAshraf, ShahidIrato, PaolaQian, LiAskalan, RandIsani, GloriaQian, PengxuAskarian-Amiri, M.E.Ishaque, AliQian, WeiAslam, MuhammadIshibashi, KeiQian, YanrongAssfeld, XavierIshii, TetsuyaQiao, KunAsterholm, I.W.Ishijima, SumioQin, YangAtanassova, RossitzaIshikawa, YasukoQuan, GuoxingAtangana, Alain R.Ishizaki, TakumaQueiroga, Cláudia S.F.Atef, EmanIshmael, Jane E.Quesada, VíctorAtkins, John F.Isidori, Andrea M.Quintana, JoséAtomi, HaruyukiIsobe, NaokiQuirino, RafaelAtrian, SilviaIsokpehi, Raphael D.Quistorff, BjørnAtsukawa, MasanoriIsshiki, YoshiakiQuock, Ryan L.Attardo, Geoffrey M.Itahana, KojiRabbani, NailaAudebert, MarcItahashi, TakashiRabilloud, F.Audenaert, KrisIto, DaisukeRachmawati, DessyAuer, ManfredIto, MichihoRadović, BranislavAuerbach, SanfordIto, TakuyaRaedschelders, KoenAuguet, TeresaIto, YasuhiroRaffaele, SylvainAureliano, M.Itoh, ToshiyukiRaftery, MartinAurora, RajeevItoh, YoshifumiRagni, LauraAustin, BrianIughetti, LorenzoRagoussis, JiannisAvarre, Jean-ChristopheIvan, RajkovicRagozin, IgorAvila, Matias A.Ivanov, A.G.Ragusa, Maria AntoniettaAvola, R.Ivessa, N. ErwinRagusa, SantoAvram, Anca M.Ivkov, RobertRahier, HubertAvvedimento, Enrico V.Iwaizumi, Masakazu G.Rahmani, MohamedAyala-Zavala, J.F.Iwamori, MasaoRajakaruna, NishantaAyalew, SahluIwamoto, SadahikoRajam, GowrisankarAyonrinde, Oyekoya T.Iwasaki, KentaRajaputra, PallaviAzad, ArunIwasaki, TomohiroRajendran, PraveenAzimzadeh, OmidIwata, JunichiRajput, NavAzmi, AsfarIzawa, HironoriRak, JanuszAzorín, FernandoIzumiya, YasuhiroRamachandran, GirishAzuma, KazuoJacob, FrancisRamakrishnan, S.Azuma, ToshifumiJacob, MelissaRaman, A.Baba, HideoJacobsen, E.E.Ramaraj, PanduranganBabbs, Charles F.Jadhav, VaibhavRamchandran, LataBabiak, IgorJahandideh, SamadRamos, JavierBabiuk, ShawnJaime, LauraRamos, SoniaBach, HoracioJaki, BirgitRamos-Solano, BeatrizBache, SørenJakočiūnas, TadasRamsden, LawrenceBachmair, AndreasJakubowski, HieronimRana, D.Backström, TobiasJalili, ThunderRandall, Stephen K.Badylak, S.F.James, AaronRandazzo, AntonioBagby, MichaelJameson, DavidRangan, VijayaBagchi, AnindyaJamieson, Stephen M.F.Rangasamy, JaykumarBagnaninchi, PierreJan, EricRanger, Christopher M.Bagnato, AlessandroJan, Tong RongRanty, BenoitBagos, PantelisJandrić, Z.Rapposelli, SimonaBai, ShuhuaJanesko, Benjamin G.Ratcliffe, GeorgeBailly, SabineJang, Byoung KukRathbone, Alex J.Bajinskis, AinarsJang, Mi-KyeongRathinavelu, AppuBaker, Mark D.Jang, SeonghoeRatner, NancyBakkeren, GuusJanitz, MichaelRattray, BenBalabaud, CharlesJanji, BassamRau, Julietta V.Balajee, AdayabalamJansen, Andrew N.Rauch, BernhardBalasubramanian, MeenaJansen, GerritRauch, U.Balaz, MilanJarret, Robert L.Rauniyar, NavinBaldessarini, Ross J.Jat, Parmjit S.Raut, SangramBalduini, WalterJaved, FawadRavegnini, GloriaBaleja, JimJaworska, NataliaRay, RatnaBalemba, Onesmo B.Jayasinghe, SuwanRay, UdayanBalestrini, Raffaellajean, LudovicRayburn, KeithBalietti, MartaJeanmart, StephaneRaychaudhuri, PradipBall, EldonJefferies, L.K.Re, SuyongBallestri, StefanoJeffery, Constance J.Reading, Benjamin J.Ballizany, WouterJeffery, GlenReal, María DoloresBallmer-Hofer, KurtJeffree, Rosalind L.Reale, MarcellaBaltazar, FátimaJellinger, KurtReason, DonaldBaluska, FrantisekJemli, SoniaRebe, CedricBamdad, CynthiaJenkins, Jill A.Rebhun, RobertBandarra, NarcisaJensen, EricRebl, AlexanderBanerjee, Hirendra nathJensen, KennethRebmann, V.Banerjee, SanjeevJensen, L.H.Reboll, Marc R.Banerjee, TaraswiJensen, ThomasReche, Pedro A.Baniahmad, AriaJeon, Tae-JoonReddy, Ashvini K.Banilas, GeorgiosJeong, DaewonReddy, M.V.Bannwarth, Markus B.Jernigan, Nikki L.Reddy, Umesh K.Bantel, CarstenJeschke, UdoRedfern, JulieBao, YupingJessel, NadiaRee, Kim MeeBao, ZhilongJi, DebinReeve, LesleyBapputty, ReenaJi, YingbiaoReeve, VivienneBaraka, KhaledJia, ChenxiRehder, DieterBarakat, AbdellatifJia, ZongchaoRehwinkel, JanBaranski, MatthewJiang, Shu-YeReich, StephenBaranzini, Sergio E.Jimi, EijiroReichel, MartinBarash, ItamarJin, ChunyangReimers, KerstinBarbarossa, I.T.Jin, TakashiReinach, Peter S.Barbera, MariagneseJing, FuyuanReis, FlávioBarberi, TizianoJobling, MalcolmReiser, Peter J.Barberon, MarieJohann, ChristophReisman, Scott A.Barbier, François F.Johannsen, DarcyReissmann, SiegmundBarbu, EugenJohnson, Hope A.Remans, TonyBarbucci, RolandoJohnson, Jason L.Remington, GaryBarbuti, AndreaJohnson, Margaret E.Rempel, L.A.Barcaccia, GianniJohnston, MichaelRen, Jian-guoBarcellos-de-Souza, PedroJohny, Manu BenRen, XuefangBarcia, Jorge M.Jolivet, RenaudRenaud, JustineBard, FredericJones, Eleanor R.Renaudineau, YvesBardoni, BarbaraJones, JohnRengo, GiuseppeBarea, E.Jones, T BuckyRenner, BertoldBarhoum, AhmedJonesa, G.T.Renner, UlrichBarlow, LindaJong, Nancy N.Renshaw, Stephen A.Barnea, Eytan R.Jonkers, DaisyRepp, JaschaBarnes, D.W.Joo, Hong-guRescifina, AntonioBarny, Marie-AnneJosé, Berna CanovasRess, Anna LenaBarolo, ClaudiaJoseph, JacobRevilla, EugenioBarr, Stephen D.Joshee, N.Rey, PascalBarrajón-Catalán, EnriqueJoshi, NikhilReyes, FernandoBarrangou, RodolpheJouline, Igor B.Reyes, José C.Barreira, JoãoJovanović, AleksandarReygaert, WandaBarresi, ValeriaJoven, JorgeReynolds, Clare M.Barreto, Maria Do CarmoJuang, Shin-hunRezaei-Ghaleh, NasrollahBarrientos, AntoniJuarranz, ÁngelesRezzani, RitaBarroso, GérardJulian, BorejdoRhee, Dong-KwonBartali, R.Julier, BernadetteRibeiro, Martha S.Bartfeld, SinaJunaid, Mohammed A.Ricciardelli, CarmelaBartoloni, GiovanniJung, Chol-HeeRichardson, AlanBartscherer, KerstinJung, Ki-HongRiddiford, Lynn M.Barzilay, Joshua I.Jung, KlausRider, Lisa G.Basaraba, Randall J.Jung, Kyung HwanRigano, DanielaBaschant, UlrikeJung, SeunhoRigano, Dott DanielaBasini, GiuseppinaJung, YoungmiRimessi, AlessandroBastola, Dhundy R.Jurk, DianaRimondini, LiaBastolla, UgoJust, SteffenRingli, ChristophBates, Shannon M.Juszczak, LesławRiou, Laurent M.Batra, JyotsnaJuzeniene, AstaRishi, Arun K.Batra, SurinderKabbaj, MohamedRissiek, BjörnBattaglia, AgatinoKaddurah-Daouk, RimaRis-Stalpers, CarrieBattino, MaurizioKadokawa, Jun-ichiRisueño, María-CarmenBauer, JohannKadota, KazunoriRitte, Jenna C. SullivanBaum, ChristelKagadis, George C.Rittner, HeikeBaum, MichelKaguni, Jon M.Rittschof, DanielBaumann, HeinzKaiser, Brent N.Riva, PaolaBaumgartner, AdiKaiser, Jean-pierreRivas, ElenaBavro, VassiliyKajiya, HiroshiRivera, VictorBayliss, SionKakisis, John D.Rivero, Rosa M.Bazhin, AlexandrKakulas, FoteiniRizzoti, KarineBeasley, SheaKalab, PetrRo, Hyeon SuBeaton, Lindsay A.Kalajdzic, PredragRobert, ClaudeBeckel, J.M.Kalendar, RuslanRoberto, DominiqueBecker, Hubert DominiqueKalinin, StanislavRoberts, AshleyBeckmann, ManfredKalinowski, JörnRoberts, Christopher J.Bédard, P.-A.Kallenbach, NevilleRoberts, Craig W.Beggs, SimonKalpaxis, Dimitrios L.Robertson, Mark J.Beier, Justus P.Kaludercic, NinaRobinson, S.M.Bejai, SaroshKalve, ShwetaRoccheri, Maria CarmelaBejan, IustinianKaminska, MartaRochaix, Jean-davidBejarano, IgnacioKaminski, Karol AdamRoche, DanielBelke, Darrell D.Kamita, GeorgeRoche, PhilippeBellastella, GiuseppeKamitani, S.Rochette, JacquesBellezza, IlariaKamo, MasaharuRödel, FranzBellincampi, DanielaKamolz, LarsRodella, Luigi FabrizioBelling, Kirstine C.Kamouchi, MasahiroRodino-Klapac, Louise R.Bello, Nicholas T.Kamunde, CollinsRodnina, Marina V.Benavente-Babace, A.Kan, LixinRodrigo, IsmaelBendahhou, SaïdKanai, MasashiRodrigues, Clara F.Benetti, FedericoKanaoka, MasahiroRodríguez, EsperanzaBenfenati, EmilioKanayama, NaohiroRodríguez, Manuel JoséBenitah, Jean-PierreKanazawa, IppeiRodriguez-Frias, F.Benito, Juan M.Kanda, TakashiRodríguez-Llorente, I.D.Benn, PeterKanda, TatsuoRogister, BernardBennet, AndrewKandror, KonstantinRoh, ChanghyunBennink, MartinKaneko, HideoRoh, HajungBenson, Al B.Kang, Bum-yongRoh, SanghoBenson, Michael D.Kang, DongchulRohs, RemoBentel, Jacqueline M.Kang, Han ChangRoidl, AndreasBentley, T. WilliamKang, HaraRolli-Derkinderen, M.Bentley-Hewitt, Kerry L.Kang, JongRoman, SabinianoBenzeroual, Kenza E.Kang, JonghoonRomano, GiovannaBerasain, CarmenKang, Kwon-KyooRomero, AlejandroBerbel, PereKang, Kyung PyoRomero, MiguelBerendsen, Floris F.Kang, Sang SunRompel, AnnetteBerendzen, Kenneth W.Kang, Taek WonRondanelli, MariangelaBerenguer, JuanKannan, S.Ropka-Molik, K.Berenguer, MarinaKanno, YuichiroRoppolo, IgnazioBergen, Martin vonKanopka, ArvydasRosa, JoaquínBerger, Joseph R.Kantharidis, PhillipRose, Aaron H.Berger, Terry A.Kanungo, JyotshnaRose, Christopher F.Berjano-Zanón, EnriqueKao, Kenneth R.Rose-John, StefanBerlien, H.-PeterKaplan, Johanne M.Rosell, RafaelBermejo-Nogales, A.Kaplan, RichardRosellini, DanieleBermúdez-Aguirre, D.Kappachery, SajeeshRosenberg, HeleneBernard-Gauthier, VadimKapur, ArvinderRosenmann, HannaBernardi, JamilaKaraca, EsraRosenzweig, DerekBernards, MattKarakasidis, T.E.Ross, Ashley E.Bernerd, FrançoiseKaralekas, DimitrisRoss, M.K.Bernhardt, Harold S.Karapanagiotis, IonnisRossi, LuisaBernier, LouisKararigas, GeorgiosRostaing, LionelBernini, RobertaKarasin´ski, JanuszRota, RossellaBernstein, Hans-GertKärenlampi, SirpaRotenberg, Susan A.Bernt, KathrinKarginov, FedorRousseau, G.Berrebi, PatrickKarkhanis, VrajeshRoutray, WinnyBerret, Jean-françoisKarlic, HeidrunRowe, Christopher L.Berron, Brad J.Karni, RotemRowland, TeishaBerruyer, RomainKarp, Effrey M.Roy, DeoduttaBerry, John P.Karpukhina, NataliaRoy, KunalBertoletti, AntonioKarras, S.N.Roy, RosaBertram, RalphKarrich, JulienRoy, SomakBerzal-Herranz, AlfredoKarstedt, S. vonRoyo, EvaBesenius, PolKarten, BarbaraRóżalska, BarbaraBetancor, M.B.Karumanchi, AnanthRozhon, WilfriedBetancur, CatalinaKasanen, RistoRuan, H.D.Betarbet, RanjitaKashiwada, ShosakuRubbi, CarlosBethanis, KostasKasper, MariaRubinow, Katya B.Bethke, GeritKassiri, ZamanehRubio, VicenteBetoret, EsterKatagiri, WataruRudat, JensBeversdorf, DavidKataoka, HiromiRudkowska, IwonaBeyth, NuritKataoka, MasatoshiRugarli, Elena I.Bhadauria, VijaiKataoka, NaoyukiRuigrok, Amber N.V.Bhamidimarri, K.R.Kataoka, YoskyRuiu, LucaBhatia, NeeharKatayama, ShigeruRuiz, Héctor A.Bhatnagar, VibhaKatiyar, Santosh K.Ruiz, T. PenaBhutani, NidhiKato, KenRullier, AnneBhuvaneswari, R.Kato, KoichiRulten, Stuart L.Bi, EnguangKato, MasayaRund, DeborahBialecka, MonikaKatsila, TheodoraRuonala, MikaBiase, Dario deKatsoris, PanagiotisRussell, Jacob A.Bidarra, SilviaKatsuma, SusumuRussell, Pamela J.Bielen, A.A.M.Kattner, LarsRusso, AnielloBierenstiel, MatthiasKauppinen, AnuRusso, NinoBiga, Peggy R.Kawabe, Jun-ichiRuthstein, SharonBigall, Nadja C.Kawaguchi, H.Ruvo, M.Bikiaris, DimitriosKawahara, AtsuoRyan, David K.Billette, ChristopheKawahara, EiRyan, Michael J.Biocca, SilviaKawai, ToshiyukiRychly, JoachimBirner-Gruenberger, RuthKawai, Vivian K.Rylott, Elizabeth L.Biron, David G.Kawakami, SusumuSabaté, JoséBisecco, AlvinoKawashima, HidekiSabatier, LaureBishop, Anthony C.Kay, EuanSabbaj, SteffanieBiswas, Pradip KKazeto, YukinoriSabbatini, SimonaBjarnsholt, ThomasKazu, KotaniSabbatino, FrancescoBjornstad, PetterKeedwell, EdwardSabotič, JericaBlais, AnneKegeles, LawrenceSacchetti, LuciaBlakley, BrianKeiderling, Timothy A.Sadee, WolfgangBlanco, Rosa M.Kellenberger, StephanSadhasivam, S.Blaner, WilliamKeller, AndreasSadik, RiadhBlankesteijn, W. MatthijsKellner, FranziskaSadiq, Muhammad W.Błaszczyk, LidiaKelly, Brian N.Sadoghi, PatrickBlázquez-Castro, AlfonsoKelly, Christopher V.Saeki, KazuhikoBleackley, Mark R.Kemp, StephanSaenen, ElineBlennerhassett, MichaelKennedy, John G.Safa, AhmadBlomberg, AndersKennedy, PatrickSaferding, VictoriaBlouin, ChristianKenyon, William J.Saggioro, DanielleBlyme, AdamKerchev, PavelSagi, SatyanarayanarajuBobryshev, YuriKerkis, IrinaSagredo, OnintzaBoccardo, FrancescoKermanshahi-pour, A.Sahoo, SushmitaBochicchio, BrigidaKern, MatthiasSahrawy, MariamBochman, Matthew L.Kersten, SanderSaid, Ahmed M.Bock, C.-T.Keshavarz, Mohsen K.Saisho, YBock, Dan G.Kessler, SonjaSaito, KuniakiBoehm, VolkerKeusgen, MichaelSaito, YoshiroBogel-Łukasil, RafałKevin, GastonSaitoh, KenjiBoger, Heather A.Keyghobadi, NushaSaitoh, NorikoBoiani, MicheleKhan, SalmaSakakibara, IoriBoisclair, YvesKhanna, NidhiSakamoto, KeiBoles, Blaise R.Khanna, SavitaSakamoto, YuichiBolmontemail, TristanKhiari, ZiedSakhaee, KhashayarBolotin-Fukuhara, Mme M.Khorram, O.Saku, TakashiBols, N.C.Khriachtchev, LeonidSakuma, KunihiroBondy, StephenKiaris, HippokratisSakuragi, YumikoBonetti, BrunoKiddle, GuySakurai, MinoruBongiovanni, LauraKiemnec-Tyburczy, K.M.Salbitani, GiovannaBonikowski, RadosławKietzmann, ThomasSaleem, MohammadBonin, SerenaKikuchi, E.Saleem, MuhammadBonner, WilliamKikuchi, HaruhisaSaleh, Fatima A.Boo, Yong ChoolKikuchi-Ueda, TakaneSalehi-Reyhani, AliBooij, LindaKim, Beom JoonSaliba, Anthony J.Boonstra, AndréKim, Chang JuSaliu, FrancescoBorgese, NicaKim, Chun HoSallé, GuillaumeBorgiani, PaolaKim, Dong JoonSalo-Ahen, Outi M.H.Borhan, Mohammed H.Kim, Dong-PyoSalsano, EttoreBorich, Michael R.Kim, Eun-CheolSalvadó, Miguel A.Bormann, JörgKim, HoonSalvatore, FrancescoBorrelli, Joseph, Jr.Kim, Hugh I.Salvi, Anna M.Bortolozzi Biassoni, A.Kim, Hye KyongSamaha, Anne-NoëlBoscá, LisardoKim, Hyeung-RakSamarasinghe, SandhyaBoscaiu, MonicaKim, Il-manSamavati, LobeliaBose, JayakumarKim, Jae YoungSamia, AnnaBoshoff, HelenaKim, Jae-jinSammons, Robert D.Bosland, PaulKim, Jae-SungSampath, PrabhaBosnjak, BerislavKim, Jin MoonSams, ThomasBoth, SanneKim, Jin-WookSamuel, MarcusBöttcher, YvonneKim, Joong SunSamuels, Mark E.Bouaïcha, NoureddineKim, JulianSanak, MarekBouaziz, SergeKim, Jung BaeSánchez Alcázar, JoséBoucheix, ClaudeKim, KiyoungSanchez, Diego H.Boughton, BerinKim, Kyoung SooSánchez, María-teresaBoulding, E.G.Kim, Kyung SooSanchez-Amat, AntonioBourgault, SteveKim, Kyung-suSánchez-Fortún, S.Bourgeois, CyrilKim, Moon KiSánchez-Pernaute, OlgaBourgeois, DominiqueKim, Na-hyungSandjo, Louis P.Boutin, JeanKim, OkjoonSandkam, Benjamin A.Bowden, ScottKim, S.J.Sandre, OlivierBowman, Teresa V.Kim, Sang-WeSandri, GiuseppinaBowness, PaulKim, SoonhagSanejouand, Yves-HenriBoyd, ChrisKim, SunghoonSangadala, SreedharaBoyer, Anne E.Kim, Sung-HoonSangelaji, BahramBoyle, Joseph J.Kim, SungtaeSanna, FabioBrader, GünterKim, SungwhanSansone, ClementinaBraga, Valdir A.Kim, Woo KyunSantamaria, RitaBraig, SimoneKim, Wun-JaeSantander, JavierBrakmann, SusanneKim, Yong SooSantarsiero, Bernard D.Brandner, SebastianKim, YongheeSantino, AngeloBraslavsky, Silvia E.Kim, YoungsooSantoni, MatteoBraun, Ralf J.Kim, Yun-baeSantoro, NicolaBravaccio, CarmelaKiminobu, SugayaSantos, CatarinaBrazzelli, ValeriaKimira, YoshifumiSantos, IsabelBrazzolotto, XavierKimura, HidetoSantos, Teresa AlmeidaBredenbeek, P.J.King, AileenSantulli, GaetanoBreitbart, HaimKing, Paul W.Sanz, LuisBreitenfeld Granadeiro, L.King-Smith, P. EwenSaravanan, PalaniappanBreitling, RainerKinugawa, ShintaroSardon, HaritzBrendlé, JocelyneKipp, Anna P.Sarkar, SibajiBrennan-Speranza, Tara C.Kira, Jun-ichiSasagawa, ToshiyukiBrenner, IngridKirby, RalphSasaki, HikaruBreton, TimothyKirchhoff, FrankSathishkumar, K.Brew, B.J.Kirschning, Carsten J.Sato, Aaron K.Breydo, LeonidKirsebom, LeifSato, KenjiBrijder, RobertKiss, István Z.Sato, NaokiBrilot, FabienneKiss, Margaret M.Sato, ShinBringmann, AndreasKiss, RobertSato, YutakaBrini, MarisaKisselev, AlexeiSatoh, ShigeruBriza, PeterKitade, YukioSatoh-Nagasawa, NamikoBrochot, EtienneKitajima, ShigetakaSaunderson, SarahBrock, GuyKitlinska, JoannaSavard, ChristianBrodmann, MarianneKittle, David S.Saviano, MicheleBroggini, MassimoKizaka-Kondoh, ShinaeSawada, KenjiroBroom, Neil D.Klaren, RachelSawalha, Amr H.Brosh, Robert M.Klein, KerstinSawers, R. GaryBrouard, SophieKliem, HeikeSayers, Thomas J.Brown, Chris M.Klintsova, AnnaScalise, MariafrancescaBrown, JamesKlitgaard, JanneScarano, ChristianBrown, Mark A.Kloiber, StefanScatena, RobertoBrown, RobertKlose, JohannesSchäfer, MichaelBrown, SusanKluza, JéromeSchaller, HubertBruce, Neil C.Knapp, StefanSchatten, HeideBruckmeier, RupertKnauer, Shirley K.Schaumburg, FriederBrudzynski, KatrinaKnetsch, Menno L.W.Scheibe, Renate J.Bruining, NicoKnölker, Hans-JoachimScheiner, SteveBrumm, Phillip J.Knoshaug, EricSchemmer, PeterBrunaud, VéroniqueKnott, RachelSchenke-Layland, KatjaBrunner, MichaelKo, Jane L.Scherer, GüntherBruno, JohnKobayashi, HiroshiSchernthaner, JohannBruserud, ØyvindKobayashi, KazuoSchernthaner, Ruediger E.Bryson-Richardson, R.Kobayashi, YoshiroSchierle, Gabriele K.Bubici, GiovanniKobeissy, Firas H.Schiller, DirkBuckley, M.Kobel, MartinSchinke, ThorstenBuckner, Jane H.Köcher, ThomasSchirrmacher, RalfBucolo, ClaudioKocmarek, Andrea L.Schlessinger, AvnerBudunova, IrinaKoda, M.Schluttenhofer, CraigBuechler, ChristaKodama, DaisukeSchmeiser, Heinz H.Bugg, Timothy D.H.Koehbach, JohannesSchmid, J.Bulla, Lee A., Jr.Koes, David RyanSchmiderer, CorinnaBullon, PedroKöfeler, HaraldSchmidt, BerndBumgardner, Joel D.Koff, AndrewSchmidt, Marco F.Buonaguro, Elisabetta F.Kofuji, KyoukoSchmidt, MarionBuono, P.Koga, FumitakaSchmidt, MortenBuonocore, FrancescoKogut, Michael H.Schmidtchen, Franz P.Bürckstümmer, TilmannKoide, HikaruSchmitz-Linneweber, C.Burg, KornelKoizumi, NozomuSchmoelzl, SabineBurger, GertraudKojima, AkikoSchneider, JoelBurgess, Catherine M.Kojima, ChieSchneider, RaphaelBurgess, Karl E.V.Kojima, TetsuyaSchoenhagen, PaulBurgoon, Mark P.Kok, KlaasSchonthal, AxelBurkhead, Jason L.Kokaze, AkatsukiSchoonbeek, Henk-janBurton, Zachary F.Kokura, SatoshiSchrader, AndreaBusch, HaukeKolehmainen, ErkkiSchraufstatter, Ingrid U.Busch, Theresa M.Kolialexi, AggelikiSchroeder, Julian I.Businaro, RitaKolovou, GenovefaSchroeter, MichaelBustamante, SoniaKolpashchikov, DmitrySchroten, CarolineBustin, Stephen A.Komine, MayumiSchrum, Laura W.Buzina, WalterKondo, EisakuSchu, PeterByrt, Caitlin SiobhanKondo, HidehiroSchuh, AlexanderCabral, JaydeeKondo, MitsuruSchüler, SusanneCaccamo, DanielaKondo, TatsuyaSchuller, Hildegard M.Cadet, FrédéricKonigsberg, William H.Schulze-Tanzil, GundulaCai, HongmeiKonishi, HiroakiSchunter, CeliaCai, LuKonstantinou, Ioannis K.Schuurink, RobertCai, WeiliKonstorum, AnnaSchwabe, Robert F.Calafiore, RiccardoKontoyianni, MeropiSchwarz, WolfgangCalani, LucaKontro, InkeriSchwarzacher, TrudeCalder, PhilipKoo, In SunSchwarzenbacher, D.Calder, PhillipKoo, Ja SeungSchweiger, WolfgangCalderaro, AdrianaKook, Koung HoonSchwertfeger, KayleeCalvano, Cosima D.Koole, Leo H.Schweser, FerdinandCalvisi, Diego F.Kooyman, David L.Sciascia, SavinoCamara, Amadou K.S.Kopczak, AnnaScobie, LindaCameli, NormaKoppang, ErlingScocchi, MarcoCammas-Marion, S.Kopra, KariScorziello, AntonellaCampbell, EwanKorach, KennethScott, NaomiCampiglia, PietroKorade, ZeljkaScott, Patrick A.Campo, Giuseppe M.Korczyn, Amos D.Scovassi, Anna IvanaCamporeale, AnnalisaKorosi, AnikoScozzafava, AndreaCampos, FranciscoKorsching, EberhardScriven, PaulCamps, JordiKoshino, HiroyukiScudiero, RosariaCancello, RaffaellaKosmacz, MonikaScurrah, KatrinaCancino, JorgeKosova, KlaraSeayad, JayasreeCanela, RamonKossida, SofiaSeca, Ana M.L.Canini, AntonellaKostin, SawaSedelnikova, Olga A.Cannata-Andía, JorgeKouadio, James HalbinSeeger, ChristophCannon, Ronald E.Koufaki, MariaSeeman, PhilipCano, AmparoKoupenova-Zamor, MilkaSeenan, ChrisCano-Delgado, AnaKouretas, DimitriosSégui, BrunoCao, LingKourist, RobertSeifert, KarlheinzCao, WilliamKourkoumelis, NikolaosSeiliez, IbanCapasso, RaffaeleKourti, AnnaSekiguchi, MihoCapitanio, NazzarenoKovar, HeinrickSelek, LaurentCaraceni, PaoloKowalczyk, MariuszSelisko, BarbaraCaragea, DoinaKowalski, KonradSempere, LorenzoCarbonell, PabloKozlowski, LukaszSen, AritroCarboni, LuciaKozyrovska, NataliaSen, NilkanthaCardarelli, FrancescoKraemer, Fredric B.Sen, ShukdebCardina, JohnKraemer, Kenneth H.Seneviratne, Chaminda J.Carè, AlessandraKraguljac, Nina VanessaSenthil Kumar, T.Carlberg, CarstenKrämer, Oliver H.Seoane Fernández, José A.Carmichael, Gordon G.Kramer, PhillipSeoane, SamuelCarneiro, ÂngelaKratimenos, PanagiotisSerantes, DavidCarrasco, PedroKraus, VirginiaSerata, MasakiCarrì, Maria TeresaKrauss-Etschmann, S.Serpente, MariaCarru, CiriacoKreader, Carol A.Serra, SerraCarter, Wayne G.Krebs, JoachimSerrano, IreneCartland, Siân P.Kreienkamp, H.-J.Serrano, Jose C.E.Carty, David M.Krenek, SaschaSerrano, MaríaCarugo, OliveroKrengel, UteSeruggia, DavideCarvalho, Ana LuisaKresanov, PetriServadei, FrancoCarvalho, JoanaKrishnan, SitaramanServi, StefanoCarvalho, LuísaKristian, TiborSestili, SaraCasaccia, P.Krol, AlainSeth, DevanshiCasarosa, SimonaKroll, JensSethi, GautamCaselli, ChiaraKronvall, ErikSevera, MartinaCasey, Theresa M.Kropski, Jonathan A.Seybold, Virginia S.Casiraghi, FedericaKrueger, R.R.Seyfert, Hans-MartinCassel-Lundhagen, AnnaKruger, RuanSgambato, AlessandroCastelein, René M.Krynetskiy, EvgenyShafi, TariqCastellano, GloriaKrystal, John H.Shah, AartiCastiglioni, BiancaKS, ChunShaio, Young-jiCastro, Antonio JesusKuan, Yu-HsiangShakibaei, MehdiCatalá, MyriamKuang, ShihuanShalin, SaraCatalano, AlessiaKubant, RuslanShamimuzzaman, MdCats, AnnemiekeKubelka, JanShankar, AnilCatts, VibekeKubina, RobertShanmugam, M.Cauli, OmarKubo, TomohikoShannahan, JonathanCavalla, P.Kuçi, SelimShapiro, BruceCavassini, MatthiasKuehl, MikeSharfstein, Susan T.Cavicchi, KevinKuerten, StefanieSharifimajd, BabakCeci, Luigi R.Kuhlmann, MarkusSharma, NaveenCeciliani, FabrizioKuiper, RolandSharma, RohitCederbaum, ArthurKuivaniemi, HelenaShavrukov, YuriCederberg-Helms, Hans C.Kukolj, GeorgeShaw, Pang-ChuiCeletti, A.Kuliawat, ReginaShe, Qing-BaiCeli, FrancescoKulka, MariannaShehu, AmardaCelius, E.G.Kumar, R.Shelomi, MatanCellerino, AlessandroKumar, SandeepShen, Jian GangCellini, FrancescoKumar, UjendraShen, JuanCervantes, Jorge L.Kumazawa, YoshinoriShen, LeslieCha, Byung-YoonKume, ToshiakiShen, Szu-ChuanCha, SangwonKumi-Diaka, JamesShen, WenCha, Tae-JoonKundakovic, MarijaShen, YipingChae, SungwookKundu, Joydeb KumarShen, Yuh-ChiangChahine, MohamedKunimasa, KazuhiroSherman, LouisChai, LijunKunisada, TakahiroShi, ChangbinCHAIMBAULT, PatrickKunnimalaiyaan, M.Shi, JiongChalak, Lina F.Kunte, HagenShi, QiangChaleix, VincentKuntz, MarcelShi, YangChalkley, RobertKunz, WolframShigeru, SatoChalmers, Jeffrey J.Kunze, GotthardShih, Chun-CheChamberlain, Jeffrey S.Kuo, Cheng-ChinShillcutt, SamuelChamberlain, StormyKuo, Hann-ChorngShim, Yhong-heeChambers, J.E.Kuo, Tzong-FuShimada, TsutomuChamcheu, Jean C.Kuramochi, KoujiShimizu, TakahikoChampion, AntonyKurasaki, MasaakiShimoda, KazuyaChamulitrat, WaleeKurata, Y.Shimono, YoshikoChan, King MingKurdowska, Anna K.Shimosawa, TatsuoChan, Koon-HoKurgan, Lukasz A.Shin, Ki-HyukChan, Kun-MingKurihara, DaisukeShin, Shyi-JangChan, Shiao-YngKurita, HirofumiShin, Ueon-sangChan, Sun OnKurose, HitoshiShinohara, Russell T.Chan, Wai-YeeKurschus, Florian C.Shintani, YasushiChandrasekar, RamanKurumizaka, HitoshiShintani, YoshinoriChang, ChengKusakabe, MakotoShiomitsu, KeijiroChang, Chien-hsingKusakabe, TakahiroShiota, MasakiChang, Chin-ChyuanKuschal, ChristianeShiozawa, MikioChang, Chiou LingKushibiki, ToshihiroShiraha, HidenoriChang, Hao-TengKusumoto, Ken-IchiShiraishi, TakehikoChang, Hsueh-WeiKutschera, U.Shirakami, YoheiChang, Hui-huaKuuskeri, JaanaShirure, VenkteshChang, Hyeun WookKuwata, Keith T.Shivapurkar, NarayanChang, Ing-FengKwa, Faith A.A.Shmizu, MasahitoChang, John P.Kwan, Joseph S.K.Shoda, MakotoChang, Jo-ShuKwok, Seung-KiShoji, SunaoChang, Long-SenKwon, Il-KeunShoji, TsubasaChang, RaymondKwon, Oh-SeungShomura, YasuhitoChang, Wei-ChaioKwon, Tae GyunShoubridge, CherylChang, Yu-JiaKwon, Young-WanShoyama, YukihiroChapel, AlainKyong, Jin BurmShrestha, Lok KumarChapman, Mark A.Kyrø, CecilieShrestha, Pravin MallaChaput, GinaKyttaris, Vasileios C.Shui, Irene M.Charlton, ClivelKyzas, George Z.Shynlova, OksanaChase, AndrewLa Rocca, Renato V.Shyu, Ann-BinChater, CasparLaederach, AlainSiah, AhmedChatonnet, ArnaudLagace, Thomas A.Sicca, FedericoChatterjee, JhinukLage, CereniusSicher, Richard C.Chaudhry, ParveshLagerstedt, Jens O.Siciliano, CarloChavez, FermanLai, Michael M.C.Siculella, LuisaChen, BinLaidlaw, Clinton T.Sidorenko, AlexanderChen, Chih-chengLakin-Thomas, Patricia L.Sieber, JonasChen, ChongyiLakka, S.Siegmund, Sören V.Chen, Chung-MingLala, Ranjna MadanSienkiewicz, AndrzejChen, ChunjungLalman, JeraldSignorini, CinziaChen, DongLam, Jenny K.W.Sigusch, Bernd W.Chen, EleanorLam, MinhSikva, Artur M.S.Chen, Guang-ChaoLaMarca, BabetteSill, HeinzChen, HouyangLambert, ElisabethSilljé, Herman H.W.Chen, Hsien-JungLambertsen, Kate LykkeSiltanen, AnttiChen, Jiann-ChuLandar, AimeeSilva, GabrielaChen, Jing-HsienLandis-Piwowar, KristinSilva, J.C.Chen, Jin-GuiLang, AndrewSilva, Luís R.Chen, Ji-YihLang, SiegmundSilva, M. Luísa S.Chen, JuanLange, Kathrin M.Silva, SnChen, Lin-ChiLangridge, PeterSilveira-Moriyama, LauraChen, MinLanham, S.A.Silvestri, ElenaChen, Peng-WenLanni, AntoniaSimal Gándara, JesúsChen, Po-YuanLaPatra, ScottSimirgiotis, Mario J.Chen, QiLapierre, PascalSimitzis, PanayiotisChen, Qi-YinLaplante, MathieuSimões, M.C.F.Chen, Ruei-mingLapres, John J.Simon, RichardChen, Szu-FuLarizza, LidiaSimon, RonaldChen, Tzong-YuehLarkin, Philip J.Simonović, MiljanChen, Wen-ChengLarkum, AnthonySimonsen, Henrik ToftChen, XiLarrainzar, EstibalizSingamneni, SaratChen, Ya-WenLarsen, Vanessa GarciaSingaravelu, G.Chen, Yen-ChouLarson, Nicholas B.Singer, Cherie A.Chen, Ying-PinLaRue, Amanda C.Singh, Ashok K.Chen, Yu WaiLash, LawrenceSingh, Chandra K.Chen, Yung-HsiangLasserre, BrunoSingh, JagdishChen, Zhe-ShengLatosińska, Jolanta N.Singh, Kameshwar P.Chen, ZhonghuaLatour, XavierSingh, KaranChen., Cheng-NanLatte, GianmarcoSingh, KashmirCheng, Fang-YuLattuada, MarcoSingh, SeemaCheng, JianlinLau-Cam, Cesar A.Singh, Udai P.Cheng, Jing-JyLaughlin, Maren R.Sinha, Krishna M.Cheng, Juei-tangLaulagnier, KarineSinnott, Michael L.Cheng, KaiLauro, M.R.Sintim, Herman O.Cheng, XingguoLausen, BertoldSirimulla, SumanCheng, XuanhongLavandera, IvanSirota, Fernanda L.Cheng, Yang-tseLavelli, VeraSirtori, Federico RiccardiCheng, YongLaVoie, Holly A.Siskind, Leah J.Cheng, ZhiliangLawrence, Clare L.Sissler, MarieCheon, Choong-illLawrence, Susan D.Sissung, Tristan M.Cheon, Yong-PilLay, Jackson O.Siu, Parco M.Cheong, Yong HwaLayden, MichaelSjogren, KlaraChermette, HenryLayden, Michael J.Skaf, Dorothy W.Chern, Ming-KaiLayh-Schmitt, GerlindeSkinner, JonathanChevalier, XavierLazzeri, ChiaraSkold, Helen NilssonChiang, Han-SunLe Borne, SylvieSkøt, LeifChiang, Tai-anLe Prell, ColleenSkripuletz, ThomasChiang, Wen-DeeLe Questel, Jean-yvesSkvortsova, IraChiappini, CiroLe, Lu Q.Slack, FrankChiappori, Alberto A.Lebleu, B.Sleebs, Brad E.Chiechio, SantinaLeboeuf, CelineSleiman, Patrick M.A.Chien, Chih-ChingLechner-Scott, JeannetteSlimestad, RuneChiesa, RobertoLeclercq, JulieSlominski, AndrzejChimenti, SergioLedesma, Maria DoloresSlovin, SusanChio, Chung-ChingLedoux, MarkSmail, Hadj-RabiaChio, In-HongLeducq, Jean-BaptisteSmart, NeilChiorino, GiovannaLee, ChangWooSmielewski, PeterChiou, Hui-lingLee, Cheng-KuoSmiesko, MartinChiou, Wen-feiLee, ChiSmirnovas, VytautasChirumbolo, SalvatoreLee, Chia-hungSmith, Alicia K.Chiu, BernardLee, Chong JaeSmith, AndrewChiu, Tsai-HsinLee, Choong HwanSmith, Anja Van BrabantChiurchiù, ValerioLee, Dae HoSmith, Clyde A.Cho, Dong-WooLee, Dong GunSmith, DarrinCho, Mi-LaLee, Duu-JongSmith, Elizabeth R.Cho, Yong-GuLee, Eun KyungSmith, Geoffrey R.Chobot, VladimirLee, Eun WooSmith, Jennifer A.Choi, Byung TaeLee, EunAhSmith, Judith A.Choi, Byung-KwonLee, GabsangSmolock, Christopher J.Choi, DonghoonLee, GsmSnyder-Cappione, J.E.Choi, Eung H.Lee, Hsin-ChenSobarzo-Sánchez, E.Choi, In-GeolLee, HueiSobczak-Thépot, JoëlleChoi, InhoLee, Hwa JinSocol, YehoshuaChoi, In-youngLee, Hye SukSodi, Anna MensualiChoi, Seok KiLee, Jai-WeiSodupe, MarionaChoi, Yoo SeongLee, Jeong I.K.Sofo, AdrianoChoi, Yung HyunLee, Juliana Tsz YanSoga, YoshimitsuChomilier, JacquesLee, Jung C.Sogorb, Miguel A.Chong, Parkson Lee-gauLee, JungwhaSoilu-Hänninen, M.H.Chopp, MichaelLee, JustinSokilde, RolfChorianopoulos, Nikos G.Lee, Kin Wah TerenceSokolova, ViktoriyaChou, C. JamesLee, KisaySoleimani, MajidChou, KuoLee, Kwang HoSoll, DieterChou, Wing-mingLee, Kwan-SungSommer, Wolfgang H.Choudhury, Swarup RoyLee, Kyu WanSomogyi, ArpadChougule, Mahavir B.Lee, Leo T.O.Son, Mi-WonChow, Cheuk Fai StephenLee, Lin-WenSone, HidekoChow, Wang-NgaiLee, Min WonSong, GuoqingChowdhury, Golam M.I.Lee, PengSong, Gwan GyuChowdhury, KamalLee, Rong-HoSong, Hae-RyongChrist, BrunoLee, Sang KookSong, JianxunChristensen, EidiLee, Sang YeolSong, JonathanChristiansen, Christian F.Lee, Sang-hanSonsalla, Patricia K.Christofidou-Solomidou, M.Lee, SanghyeobSoon, Yu YangChristov, Christo Z.Lee, Sang-WonSoreq, HermonaChrzanowski, ŁukaszLee, Seong-RyongSoriani, OlivierChu, XiangpingLee, Shyi-LongSoriano, ElenaChuang, Wan-LongLee, Sung ChulSoric, AudreyChueh, Pin JuLee, TaeseungSotriffer, ChristophChun, Heung JaeLee, Tzong-shyuanSouders, Colby A.Chun, JeroldLee, Vincent WsSouza-Smith, Flavia M.Chung, EuiryongLee, Wing-keeSovak, GuyChung, Wen-HungLee, Won SupSpagnuolo, AntoniettaChung, Ying-ChienLee, Won-HaSpagnuolo, CarmelaChwalibog, AndréLee, WooinSpalletta, GianfrancoChyau, Charng-CherngLee, WooyoungSpandou, EvangeliaCiaccio, MarcelloLee, Yoon KwangSpano, GiuseppeCiappellano, SalvatoreLee, YoungSparks, Paul B.Ciardelli, GianlucaLee, Yu-HsiangSparrow, Janet R.Cichero, ElenaLeffler, JonatanSparvoli, FrancescaCicuta, PietroLefterov, IliyaSpelman, LyndaCillo, ClementeLegendre, LaurentSpengler, UlrichCindrova-Davies, TerezaLegouin-Gargadennec, B.Spiess, ChristophCiofani, GianniLehmann, Alan R.Spinazzola, AntonellaCirillo, GiuseppeLehmann, ChristianSpitzner, MelanieCirone, M.Lehto, TaaviSpizzo, GilbertCitron, Bruce A.Lei, XiaSpoelder, JanClark, ShannonLeigh, Spencer A.Sredni, Simone T.Clarke, AnthonyLeitão, AnaSrinivas, KeerthiClaudia, Grothe, P.Leitz, ThomasSrivatsan, MalathiClaudia, VolpiLembo, SerenaSrivenugopal, KalkunteClaus, HaraldLEN, ChristopheStaege, Martin S.Clavel, Marie-AnnickLenaz, GiorgioStaff, Anne CathrineClawson, Gary A.Lennartsson, Patrik R.Stamatatos, Theocharis C.Clem, Brian F.Lenon, GeorgeStamelos, VasileiosClément, GillesLeonard, NolaStammler, GerdClementi, NicolaLeone, AlessandroStanley, JoannaClendenen, Tess V.Leong, David TaiStansfield, KirstieCleveland, Beth M.Leopold, Jane A.Stanta, GiorgioCloos, JacquelineLephart, Edwin D.Stanton, Robert C.Coates, Christopher J.Leppla, Stephen H.Starostik, PetrCobb, JoannaLerman, LilachStassi, GiorgioCocetta, GiacomoLessard, ChristopherStaudacher, Alexander H.Cockerill, GillianLessman, CharlesStead, Lucy F.Codacci-Pisanelli, G.Leucht, S.Steardo, LucaCoelho, José A.Leung, Chung-HangStec, David E.Cognasse, FabriceLeung, DavidSteen, Andrew D.Cohen, AdamLeung, Ping-ChungSteenwijk, Martijn D.Cohen, YoramLeung, Yuet-KinStefanidou, Maria E.Cohick, WendieLeuven, Fred VanStefano, CassanelliCohney, SolomonLevine, Andrew J.Stefano, CescoColbert, Robert A.Levraud, Jean-pierreSteffens, BiankaCole, SteveLewandowski, BartoszStehouwer, Peter PaulColeman, DavidLewin, AlSteigedal, Tonje S.Coleman, William B.Lewinson, OdedSteinritz, DirkColes, Alasdair J.Lewis, MichaelStepensky, DavidColl, JosepLewis, RamseySteuer, Andrea E.Colle, IsabelleLewkowich, Ian P.Stevanato, PiergiorgioColliec-Jouault, SylviaLey, Alexandra C.Stevens, StanleyCollins, Helen M.Ley, SteveStevenson, GraemeColombié, SophieLeygue, EtienneStiborova, MarieColombo, C.Leyrat, CedricStich, StefanColuccia, E.Lezot, FrédéricStiegelbauer, VerenaConcas, AlessandroLi, BingStocco, GabrieleConcordet, Jean-PaulLi, ChiStock, PeggyConlon, Michael A.Li, DefangStockmann, RegineConstanti, MagdaLi, GuoshengStoddard, Barry L.Conte, PierfrancoLi, Hsing-HuiStoecklin, GeorgConti, ArioLi, JianmingStone, Trevor WilliamContini, AlessandroLi, JinsongStope, Matthias B.Cook-Mills, Joan M.Li, Kuo-BinStournaras, ChristosCooper, ArthurLi, LeiStrehmel, NadineCooper, William T.Li, LiStricker, SigmarCoppin, HélèneLi, LingStrom, Jakob O.Coppola, David M.Li, Mei-LingSu, Chun-LiCoppola, NicolaLi, MingSu, ChunmingCorcoran, Brendan M.Li, QinghongSu, Dong-MingCordero, MarioLi, Quan-ZhenSu, HuaCordero, PaulLi, RonaldSu, Jui-HsinCorey, Seth J.Li, SihengSu, Tsung-PingCorley, M.M.Li, TiangangSu, Tung-HungCorporeau, CharlotteLi, Wen-hongSu, Wen-DaCorreia, Sónia C.Li, XiaohuaSu, YeuCortes, Andres J.Li, XiaohuiSuárez, JuanCortez, M.M.Li, XiaoliSuenaga, HikaruCos, PaulLi, Xudong JoshuaSuetsugu, ShiroCosentino, UgoLi, XueweiSugawara, AkiraCosgrove, DanielLi, YefuSugawara-Narutaki, AyaeCosma, PinalysaLi, YiSugimoto, YoshiakiCossigny, DavinaLi, YongSugimura, HaruhikoCosta, ValerioLi, YongfangSugita, NorikoCostantini, DavidLi, Yu-youSugiyama, HiroshiCosta-Rodrigues, JoãoLian, Christine G.Suh, Dae-yeonCostoya, Jose A.Lian, Jane B.Suh, Hyung JooCôté, Jean-FrançoisLiang, Xue-HaiSuh, Pann-GhillCotella, Dott DiegoLianidou, Evi S.Suhling, KlausCoto, MaireneLiao, Jiunn-wanSukumaran, AnilCouffin, Anne-ClaudeLiao, Jyh-FeiSullivan, Shannon D.Couttet, PhilippeLiao, PingSuman, Devi S.Couture, RéjeanLibert, SergiySumby, KristaCova, LucynaLiebisch, GerhardSumi, ShoichiroCovès, JacquesLiehn, Elisa A.Sumich, AlexCragg, Peter J.Liekens, SandraSumner, KatieCrago, JordanLienkamp, KarenSun, DandanCraig, Paul M.Liermann, Johannes C.Sun, GenlouCrescioli, ClaraLim, DongwookSun, Raymond Wai-YinCreusot, NicolasLim, Jong-SeokSun, WeiminCrews, ColinLim, Keng GatSun, YingCrisanti, AndreaLim, Kyung-MinSun, ZhonghuaCritchley, JuliaLim, Siew PhengSundin, GeorgeCroce, FaustoLim, Sung-JigSung, Ping-JyunCronin, MarkLim, Young-CheolSung, SibumCrossman, David J.Lima, RenataSunseri, FrancescoCrosthwaite, Susan K.Limam, FeridSupiot, StéphaneCruchaga, CarlosLin, Cheng-WenSupowit, Scott C.Csuk, RenéLin, Chi-ChenSupuran, Claudiu T.Cueto, MercedesLin, Chih-ChienSurai, PeterCui, JunLin, Chiou-fengSureshkumar, Kalathil K.Cullis, ChristopherLin, GlennSurette, Marc E.Culman, JurajLin, HoSurguchov, AndreiCurci, John A.Lin, Hung WenSuter, Marc R.Curiel, LauraLin, HungduSuter, Melissa ACurley, Gerard F.Lin, Hung-YinSutherland, John B.Czosnyka, ZofiaLin, KuiSuyama, MikitaDa Silva, Douglas GaniniLin, Kwang-HueiSuzuki, HitoshiDa Silva, M.L.AlvesLin, LiSuzuki, KaoruDa Silva, Mateus WebbaLin, Ping-TingSuzuki, MasatakaDacher, MatthieuLin, QinsongSuzuki, NoboruDaglia, MariaLin, QiongSvensson, Katrin J.Dai, TianhongLin, QishengSvircev, Antonet M.Dai, WenhaoLin, ShuhaiSwahari, VijayDai, ZhaohuaLin, Shu-pingSwain, MartinDai, ZongLin, Shyh-HsiangSwales, CatherineDaiber, AndreasLin, Wan-wanSwaminathan, SubramaniDakhama, AzzeddineLin, YongjunSwarts, Benjamin M.Dalbies, RozennLin, Yun-lianSweet, KendraD'all aqua, StefanoLindeman, JanSweetman, DylanDalmeijer, GerdienLinder, StigSwevers, LucDalrymple, Brian P.Lindholm, DanSwiecicki, Jean-MarieDamalas, ChristosLindholm-Perry, A.K.Swindle-Reilly, Katelyn E.Danby, F. WilliamLindner, DanielSykiotis, Gerasimos P.Dane, FennyLindqvist, PerSzabò, IldikòDanesi, FrancescaLindsay, Christopher D.Szarvas, T.Danser, JanLinert, WolfgangSzczubiałka, KrzysztofDarabi, HatefLing, Maurice HtSze, Stephen Cho WingDarder, MargaritaLing, Xuefeng B.Taanman, Jan-WillemDare, AndrewLing, Yong-ChienTabata, YasuhikoDarvin, MaximLionetti, LillàTada, HayatoDas, AninditaLiou, Ying-MingTada, YuichiroDas, ChittaranjanLipinski, P.Taddei, Maria LetiziaDas, RajLipkowski, JacekTafi, AndreaDas, SamarjitLipton, Jeffrey I.Tågerud, SvenDas, Subha R.Litescu, Simona C.Taglietti, AngeloDas, SumanLitinas, Konstantinos E.Tago, MegumiDasmahapatra, AsokLiu, Chen-HuaTaguchi, YoshihiroDave, Kunjan R.Liu, DelongTaheri, ArashD'Aversa, Teresa G.Liu, FengTahtamouni, Lubna H.David, JonathanLiu, Fwu-HsingTain, You-LinDavid-Cordonnier, M.-H.Liu, Hui-HsuanTaipaleenmäki, HannaDavide, Degli EspostiLiu, JianTaira, JunseiDavidson, Sean M.Liu, JinTajbakhsh, J.Davidson, William S.Liu, JingwenTajima, KenjiDavies, Thomas G.E.Liu, LeyuanTajiri, NaokiDavis, Gregory D.Liu, LiangTakagi, KiyoshiDavis, Michael E.Liu, ManTakahara, MitsuyoshiDavis, Mindy I.Liu, RuiqiangTakahashi, KazuhiroDay, AndrewLiu, ShujingTakahashi, WataruDay, ReginaLiu, Wang-TaTakahisa, KanekiyoDayal, SanjanaLiu, WeiTakaki, AkinobuDayan, DanLiu, XiaoyanTakami, SeiichiDe Berardis, DomenicoLiu, YanqunTakami, TaroDe Bernonville, T.D.Liu, YingTakaoka, AkinoriDe Braud, F.Liyana-Arachchi, T.Takashi, YagiDe Bruin, ChristiaanLizard, GérardTakeda, YouheiDe Carolis, CaterinaLlorente, BriardoTakehana, YusukeDe Feo, VincenzoLo, Huang-MuTakekoshi, SusumuDe Geest, BartLo, Mindy S.Takemori, HiroshiDe la Campa, Adela G.Lobodin, Vladislav V.Takenaka, YasuhiroDe la Escosura, AndrésLocatelli, MarcelloTakeshita, FumitakaDe la Fuente, HelenaLockridge, OksanaTakeshita, HaruoDe la Fuente, Jesus M.Lodola, AlessioTakeuchi, HideyukiDe la Fuente, LeonardoLoh, Amos Hong PhengTakeuchi, YasuDe la Mata, JavierLomakin, Ivan B.Takeuchi, YukoDe la Monte, SuzanneLombardi, PietroTakumi, ShigeoDe la Serrana, Daniel G.Lonardo, AmedeoTakuro, NakayamaDe Lange, PieterLondon, NirTalbert, Joey N.De Levie, RobertLonez, CarolineTamura, MasaakiDe Lorenzo, AntoninoLong, AudreyTan, Dun-XianDe los Santos Valladares, L.Longley, D.B.Tanabe, M.De Lourdes Pereira, MariaLoo, Ruey-LengTanabe, YooDe Pedro, NuriaLood, ChristianTanaka, HiromiDe Peppo, Giuseppe M.López-Atalaya, José P.Tanaka, HiromitsuDe Polo, AnnaLopez-Hellin, JoanTanaka, HiroyukiDe Steur, HansLopez-Llorca, LuisTanaka, KohichiDe Vendittis, E.Lopez-Vaamonde, CarlosTanaka, MinoruDe Visser, S.P.Lord, Megan S.Tanaka, NaokiDealy, Caroline N.Lotrich, FrancisTanaka, NaonobuDean, Zachary S.Lötsch, JörnTanaka, TakujiDeans, Andrew J.Lou, Bih-ShowTanaka, Tim HideakiDearden, John C.Lou, Jie-ChungTanavde, VivekDeCarlo, Arthur A.Lou, Xiang-YangTang, WeiDecker, Brian S.Louime, CliffordTang, WenlongDedoussis, George V.Loura, Luís M.S.Tang, XiaojiaDeFilippis, Victor R.Løvdal, TrondTani, TokioDegregorio, Michael WLovell, MarkTanigawa, TetsuyaDeharo, EricLu, Chi-YuTanimizu, NaokiDehouck, YvesLu, HongTanner, N. KyleDekker, MarloesLu, JianTanoi, KeitaroDel Carpio-Perochena, A.Lu, JinjianTanonaka, KouichiDelerue, FabienLu, JunTanou, GeorgiaDeleuze, MichaelLu, LinchaoTao, LinDelgado Olguín, PaulLu, Ming-WeiTao, YaxiongDelivoria-Papadopoulou, M.Lu, PaulTarantino, GiovanniDemir, Ihsan EkinLu, Rong-RongTarozzi, AndreaDemmerle, JustinLu, Tse-MinTarro, GiulioDemorrow, SharonLu, WTartaglia, Gian GaetanoDemyanets, SvitlanaLuben, TomTasciotti, EnnioDen Dunnen, Wilfred F.A.Lucassen, Paul J.Taskinen, SeppoDen Hertog, JeroenLucchin, MargheritaTate, Rothwelle J.Denizot, B.Luco, Reini FTatsumi, KazuyaDenoyer, DelphineLudwig, RolandTatullo, MarcoDenti, MichelaLudwig-Müller, JuttaTaupitz, MatthiasDepalma, Ralph G.Lui, EdTava, AldoDerks, Terry G.J.Lui, Vincent Chi-HangTavazzi, BarbaraDeshayes, SébastienLum, Julian J.Tawata, ShinkichiDesideri, AlessandroLuna, AurelioTayabali, AzamDesmarets, ChristopheLuna, DiegoTaylor, Larry T.Desmet, TomLunel-Fabiani, FrançoiseTaylor, Maureen E.Dettori, Maria LuisaLuo, PeigaoTaylor, Molly A.Deurwaerdère, PhilippeLuo, Zhong-ChengTaylor, Nicolas L.Devergne, OdileLupiáñez, José A.Taylor, Peter W.Devesa, J.Luthey-Schulten, ZaidaTaylor, Richard W.Dewi, MewahyuLuzhkov, Victor B.Tazaki, HiroyukiDey, MoulLyng, Fiona M.Tchapla, AlainDhandapani, Krishnan M.Lyng, HeidiTcholakova, SlavkaDhawan, SangeetaLysaght, JoanneTeeri, Teemu H.Dhondt-Cordelier, S.Ma, ChaozhiTeets, Nicholas M.Di Cesare Mannelli, L.Ma, DaqingTegeder, IrmgardDi Giulio, MassimoMa, Dik-LungTegler, G.Di Luccia, AldoMa, GuojiaTehranchian, P.Di Paolo, ThérèseMa, Jin YeulTehrani, Kourosch A.Di Primio, R.Ma, LiangTeijido, OscarDi Renzo, LMa, WeiTeissie, JustinDi San Lio, Gaetano M.Ma, YongjieTejero, JesúsDi, RongMa, YuelongTen Cate, HugoDiamond, BettyMacDiarmid, Colin W.Tenbrock, KlausDias, CarlosMace, ThomasTéoulé, EvelyneDíaz, JoséMachiels, KathleenTerachi, ToruDiccianni, Mitchell B.Macias Sanchez, Antonio J.Teran, Francisco J.Díez, DavidMacías, PedroTerao, KimioDijkstra, Bauke W.MacIntosh, Gustavo C.Teschke, RolfDikalova, AnnaMacManes, MatthewTesli, MartinDing, YuchuanMacPhee, Iain A.M.TESSARO, DavideDinh, Dzung H.Macqueen, Daniel J.Testai, Fernando D.Dini, LucianaMadej, ThomasTetard-Jones, CatherineDinis, TeresaMaeda, TatsuyaTetsu, OsamuDiot, ChristianMaehr, RenéTettamanti, GianlucaDioum, Elhadji M.Maestro, MiguelTevosian, Sergei G.Dittrich, MarcusMagata, YasuhiroTewari-Singh, NeeraDixon, DanMaggioni, DanielaTfayli, AliDixon, David A.Magid, Abdulmagid A.Thaler, LeaDiz, Debra I.Magnaldo, ThierryTharakaraman, KannanDmitriev, PetrMagnus, PerTheile, DirkDo, Yi-yinMagyari, MelindaThein, Tun-LinnDobashi, YohMahasenan, KiranTheodoly, OlivierDobens, Leonard L.Maher, Christopher A.Theodoropoulos, JohnDoerr, MarkusMaher, PamelaThevelein, Johan M.Doerrler, WilliamMahimainathan, LeninThèvenod, FrankDoi, HideyukiMahjoub, M.-S.Thibaudeau, SebastienDolinsky, Vernon W.Mahmoud, Soheil S.Thilmony, RogerDomingos, Pedro M.Maicas, SergiThiriet, MarcDominguez, Gustavo A.Maire, PascalThoma, ChristianDonaghue, JacquiMaisch, TimThomas, Helen E.Dondi, DanieleMaitre, MichelTHOMAS, OlivierDoniach, SebastianMajumder, MousumiThomas, Olivier P.Donnelly, Ryan F.Mak, AnselmThompson, Deborah J.Donno, DarioMak, Nai K.Thompson, JenniferDonohoe, Bryon S.Maki, KosukeThompson, Mary KathrynD'orazio, JohnMakinodan, ManabuThompson-Snipes, L.Dorea, JoseMalaguarnera, MicheleThomsen, GeraldDosio, FrancoMalavasi, FabioThor, Ann D.Dosztányi, ZsuzsannaMalchiodi-Albedi, F.Thorin-Trescases, N.Douglas, Jack F.Malde, AlpeshThornburg, KentDouglas, Timothy E.L.Malemud, Charles J.Thrasyvoulou, AndreasDoulias, P.-T.Malemud, Charles J.Threlkeld, StevenDouni, EleniMalheiro, RicardoTian, XiaoyuDowd, Patrick F.Mallipeddi, Prema LathaTian, YingfangDoyle, SiamsaMalmsten, MartinTichauer, KennethDrakoulis, NikolaosMancuso, PeterTielens, LoiusDressel, AlexanderMandell, Samuel PierceTijssen, PeterDriessen, ChristophManenti, RaoulTikoo, Suresh K.Drobizhev, MikhailManfredi, GiovanniTilkorn, Daniel-johannesDrosten, M. DrostenMang, Thomas S.Timko, Michael PaulD'Souza, ManoranjanManga, PrashielaTimmerman, PeterDuan, YuyouMangialasche, FrancescaTimmons, LisaDuarte, Filipe V.Mannell, HannaTimmusk, SalmeDuarte, MargaridaManning, ThomasTiozzo, StefanoDuarte, T.L.Manolson, Morris F.Tirone, FeliceDube, Dipak K.Manoranjan, BranavanTitica, MarianaDübel, StefanMansoori, G. AliTitorenko, Vladimir I.Dubey, Mukesh K.Månsson, AlfTivendale, Nathan D.Dubey, Raghvendra K.Mantero, SaraTiwari, SanjayDubielecka-Szczerba, P.Manuella, RiganoTobajas, MontserratDubini, AlexandraManukjan, GeorgiTobiume, KeiDubrova, YuriManz, Rudolf ArminTobon, Yeny A.Duda, KatarzynaMarasco, DanielaTognetti, VincentDudás, JózsefMarc, IsabelleTognini, PaolaDufès, ChristineMarcacci, MaurilioTokars, Valerie L.Duhamel, JeanMarcelli, AugustoToledano, ManuelDumas, RenaudMarchenko, OlgaTolmachev, VladimirDumez, SylvainMarchesan, SilviaTomasch, JürgenDumon, S.Marchetti, PhilippeTomasi, NicolaDumont, MarcMarco, CarmenTombini, M.Dunbar, Gary L.Marcos, MercedesTomiyasu, HirotakaDuncker, DavidMarcu, Kenneth B.Tomlinson, Gail E.Dundas, IanMarcucci, FabrizioTommassino, MassimoDunham, ShariMaréchal-Drouard, L.Tomofuji, TakaakiDunn, LouiseMarinaki, A.M.Tomonaga, KeizoDurand, Jean-OliverMarino, AngelaTompa, PeterDürrwald, RalfMariottini, GianluigiTonda-Turo, ChiaraDusso, AdrianaMarison, IanTong, LingyingDustin, Lynn B.Markou, GiorgosTong, StephenDvornyk, VolodymyrMarmiroli, NelsonToniolo, AntonioDwivedi, ChandradharMarondedze, ClaudiusTonks, Nick K.Dytfeld, JoannaMarques, FernandaTonnabel, JeanneDzamko, NicolasMarques, Isabel PaulaTopisirovic, IvanDziubla, ThomasMarques, Paula A.S.S.Tordjman, SylvieEbner, NicoleMarsault, EricTori, MotooEbrahimi, Kourosh H.Marshall, Gailen D.Toropov, Andrey A.Eckardt, KristinMarsillach, JuditTORRENS, FranciscoEckl, K.M.Martel, AnTorres, J. AntonioEder, Iris E.Martel, FátimaTorres, JosemaEfird, JimmyMartel, S.Tortora, P.Efrat, HimonMartens, PennyTosti, S.Eftekharpour, EftekharMartí, SérgioTouraud, DidierEggert, JullieMartín, A.Touyz, Louis Z.G.Eggler, AimeeMartin, F.Tovit, RosenzweigEglin, DavidMartin, JuliaToyoda, AtsushiEgloff, Ann MarieMartin, Kathleen A.Trabace, LuigiaEhrhardt, AnjaMartin, Sara L.Trabocchi, AndreaEhrlich, HermannMartin, Todd M.Trabolsi, AliEid, NabilMartín-Aragón, SagrarioTrachtman, HowardEisel, UlrichMartín-Cordero, CarmenTraktuev, Dmitry O.Elati, M.Martinelli, AdrianoTrendowski, MatthewEleouet, Jean-FrançoisMartinelli, fillopoTrenfield, Melanie A.El-Gendy, Ahmed A.Martinez, Anne-sophieTretyakova, Natalia Y.Elhanafi, Ahmad OmarMartínez, ConstantinoTrevisan, AndreaElhendy, AbdouMartinez, ManuelTriguero, IsaacElisabetta, DonzelliMartínez, Miguel A.Trindade, HelenaEljaafari, AssiaMartinez, VicenteTripathi, PrateekElke Holinski-Feder, ElkeMartínez-García, Pedro J.Tripodi, MarcoElkhayat, Ehab S.Martínez-González, JoséTrischitta, FrancescaEllervik, ChristinaMartinez-Orgado, J.Trollmann, ReginaEllis, Ronald E.Martínez-Sánchez, NoeliaTrollope, AlexandraElosua, RobertoMartino, SabataTromp, GerardElshimali, Yahya I.Martins, L. MiguelTrott, Josephine F.Emerson, JosephMartinusen, DanTrotta, Christopher R.Emond, M. PatrickMaruf, Abdullah AlTrouillas, PatrickEnari, MasatoMarverti, GaetanoTrovato, GuglielmoEndersby, N.M.Marzocco, StefaniaTrucco, MassimoEnglish, NiallMasaaki, Konishi MasaakiTrujillo, GlendaEo, Seong KugMasaki, HitoshiTryfonidou, Marianna A.Eppard, ElisabethMasci, StefaniaTsaftaris, AthanasiosEpperly, MichaelMashek, DouglasTsai, C.Y.Ericson, Marica B.Masi, AntonioTsai, Cheng-FangEriksen, Niels ThomasMasoero, GiorgioTsai, Eing-MeiErtani, AndreaMason, D.L.Tsai, Fuu-JenEscames, GermaineMasotti, AndreaTsai, Henry P.Escolà-Gil, Joan CarlesMasquida, B.Tsai, Hui-Hsu GavinEsguerra, Jonathan Lou S.Masters, Colin L.Tsai, Keng-changEspen, RimstadMastrangelo, Anna M.Tsai, Shau-WeiEspinosa-Urgel, ManuelMasuda, HaruchikaTsai, Shih-ChangEssani, KarimMasuda, KiyoshiTsai, SientangEsteban, Maria AngelesMasuda, MichiakiTsakalof, A.Estelrich, JoanMatar, Samir F.Tschon, MatildeEsteve, JordiMatés, José M.Tsikas, DimitrosEsteve-Romero, JosepMatesic, Diane F.Tsiplakou, EleniEsteves, Ana CristinaMateus, CatarinaTsitsilonis, Ourania E.Ethirajan, AnithaMateus, NunoTsonis, Panagiotis A.Evans, Bradley S.Matile, StefanTsuji, PetraEvans, David R.Matoba, NobuyukiTsuji, ShojiEvans, Joseph L.Matos, Ana RitaTsukiyama, TakujiEve, DavidMatros, AndreaTsunoda, IkuoEvensen, ØysteinMatsubara, JoanneTsutsui, ShigeyukiEwart, Kathryn VanyaMatsubara, KeiichiTu, Shi-MingExner, ThomasMatsugo, SeiichiTubaro, CristinaExpert, DominiqueMatsui, ToshiroTuck, Astrud R.Eykyn, Thomas R.Matsunuma, S.Tudisco, RaffaellaEynde, Jean J. VandenMatsuoka, DaisukeTuggle, Christopher K.Ezura, YoichiMatsuoka, MasatoTulliani, Jean-MarcFabiani, RobertoMatsuoka, Shin-ichiTuma, PamelaFabio, VianelloMatsusaki, MichiyaTung, Bui ThanhFacelli, Julio C.Mattioli-Belmonte, M.Tung, Tao-HsinFadl, AminMattoo, AutarTurco, GianlucaFagerstedt, Kurt V.Mattsson, JimTuriel, MaurizioFahlman, Richard P.Matyas, JessicaTurkbey, B.Faísca, PatríciaMaunoury, V.Turng, Lih-ShengFalahati-Anbaran, M.Mavragani, Clio P.Turunen, Mikko P.Falasca, M.Mavrogonatou, EleniTuttolomondo, AntoninoFan, XiuchengMayer, ClemensTyagi, NeetuFang, WenwenMayhan, William G.Tyner, Jeffrey W.Fang, XianglingMaytin, Edward V.Typas, Milton A.Fanizzi, FrancescoMazière, CécileTyurina, Yulia Y.Faoro, FrancoMazzucotelli, ElisabettaTyynismaa, HennaFaraloni, CeciliaMcArdle, HarryTyystjärvi, TainaFarinos, H. MerleMcArthur, J. VaunTzang, Bor-ShowFariselli, PieroMccarthy, John J.Tzetis, MariaFarren, MatthewMcClung, C.A.Ubertini, FilippoFata, Giorgio LaMccullough, MichaelUchida, ShizukaFaul, ChristianMcCullumsmith, R.E.Uchida, YoshikazuFazio, Pietro DiMcCully, James D.Ueda, NatsuoFedele, FrancescoMccully, Kilmer S.Uegaki, KoichiFehling, HeatherMcDonagh, BrianUehara, TomoyaFei, LikunMcdonald, CourtneyUeno, Shu-ichiFeichtinger, Georg A.Mcdougall, Gordon J.Ufer, ChristophFeinendegen, LudwigMcFarland, SherriUhal, Bruce D.Feizi, SoheilMcGrath, Patrick T.Uhde-Stone, C.Felby, ClausMcgrew, Michael J.Ulisse, SalvatoreFelder, EduardMcGuffin, Liam J.Ullah, AshikFeliers, DenisMcHenry, PeterUllah, HemayetFelix Saavedra, Maria JoséMcintosh, E. David G.Umemura, MycoFelli, Isabella C.Mcintosh, RobertUmemura, ShigekiFelmlee, Daniel J.McKinney, Garrett J.Uppugunduri, C.R.Feng, ChangjianMcLachlan, GerryUrabe, DaisukeFeng, DanMcLaughlin-Drubin, M.Urbanc, BrigitaFeng, XiqiaoMcMahon, Hilary E.M.Ureña, EnricFeng, YangboMcMahon, Lawrence P.Ushio-Fukai, MasukoFeng, YibinMcMahon, PeterUtashima, YuuFeng, Z. VivianMcmurray, David N.Uus, KaiFenoglio, ChiaraMcNamara, Coleen A.Uversky, VladimirFenoll, CarmenMedeiros-Domingo, A.Uversky, Vladimir N.Fenwick, Mark A.Medina Piles, VicenteVacca, AngeloFeo, FrancescoMedina, Francisco JavierVago, RiccardoFeola, MauroMeeker, Rick B.Vaira, ValentinaFerlizza, EneaMeharg, CarolineVaisar, TomášFernandes, Emanuel M.Mehboob, ShahilaVaisburg, ArkadiiFernandes, Susana C.M.Mehdizadeh, MehdiVajro, PietroFernandez, JessieMehr, ShaadiVale, PauloFernández, José J.Mehta, Akul Y.Valencia, C. AlexanderFernández-Bolaños, JuanMeijles, DanielValencia, GregorioFernandez-Carvajal, AsiaMeiser, PeterValenciano, Ana IsabelFernandez-Garcia, MartaMelendy, TomValenzuela, ManuelFernandez-Lafuente, R.Mélinon, PatriceValero, RosendoFernández-Ponce, M.T.Melkani, G.C.Valli, VeronicaFernández-Ruiz, JavierMells, Jamie E.Valone, Steven M.Feron, KrishnaMelo, Eduardo P.Valtueña, Francisco J.Ferraioli, GiovannaMeloni, RolandoValverde, Ángela M.Ferrand, JonathanMelzig, MatthiasVan Coppenolle, FabienFerrante, AntonioMemelink, JohanVan Dam, MikeFerrarese, CarloMencherini, TeresaVan Damme, JoFerrarini, AlbertoMendes, EduardaVan de Donk, Niels W.C.J.Ferrazzi, EnricoMendoza-Cózatl, D.G.Van De Sluis, BartFerreira, A.F.Menendez, RosaVan Den Bergh, HubertFerreira, DanielMeng, XiangbingVan den Meiracker, A.H.Ferreira, João R.Meng, YizhiVan den Oord, Stijn C.H.Ferreira, ThierryMénoret, SéverineVan der Kuyl, AntoinetteFerrini, SilvanoMentzer, Steven J.Van der Lee, RobinFetzner, SusanneMercer, CarolVan Der Meer, PeterFielding, JoanneMercer, Julian F.B.Van Der Straaten, TaharFierabracci, AlessandraMerkel, OlafVan Der Voet, GijsbertFindlay, Victoria J.Merlino, AntonelloVan Duijn, BertFinelli, CarmineMerlot, SylvainVan Dyke, KnoxFiocco, DanielaMetheny-Barlow, Linda J.Van Dyke, Mark E.Fiona, LynchMetherell, LouiseVan Kessel, Ad GeurtsFiorina, PaoloMetzinger, LaurentVan Lieshout, LisetteFirer, MichaelMeyer, Barbara J.Van Oers, NicolaiFirestone, GaryMeyer, DeniseVan Veldhuizen, PeterFischer, Hans-MartinMeyer, EtienneVan Vliet, Sandra J.Fitton, HelenMeyer, FlorenciaVan Wolfswinkel, J.C.Fitzgerald, Rebecca C.Meyer, Hans-PeterVanakker, OlivierFlisikowska, TatianaMeyerholz, DavidVanamala, JairamFlorea, Ana-MariaMezzenga, RaffaeleVanarotti, MurugendraFlorio, TullioMiao, JunVandamme, DriesFlower, AndrewMicali, NorbertoVandenbussche, FilipFluhr, RobertMiccadei, StefaniaVanella, LucaFlynn, AidanMicheau, OlivierVanHorssen, JackFoderà, VitoMichelhaugh, Sharon K.Van't Hof, Arnoud W.J.Fomenko, Dmitri E.Michels, Alexander J.Vantarakis, ApostolosFondevilla, SaraMichetti, FabrizioVaporidi, KaterinaForbes-Hernandez, T.Y.Michiels, CarineVarela-Ramirez, A.Ford, Lance P.Mico, Juan A.Varghai, DavoodForest, Jean-ClaudeMidde, KrishnaVarra, MichelaForhead, Alison J.Midgley, AdamVarrot, AnnabelleFormisano, PietroMielenz, ManfredVashee, SanjayForster, BrittaMigita, SatoshiVasiljevic, TodorForthun, Rakel BrendsdalMigliaccio, Anna RitaVassalle, CristinaFoster, Leonard J.Migliore, LuciaVassilopoulos, GeorgeFosu-Nyarko, JohnMiguel, GraçaVaudry, DavidFoti, Mario C.Mikami, ToshinariVaughan, GilbertoFotopoulos, VasileiosMiki, YasuhiroVaughan, Roger A.Fouilland, EricMilà, MontserratVavvas, Demetrios G.Foulds, CharlesMilde-Langosch, KarinVaz, Deisi AltmajerFournier, ClaudiaMildner, MichaelVecchione, CarmineFox, Keith R.Milella, LuigiVecoli, CeciliaFrancino, PilarMilhinhos, AnaVedani, AngeloFrancis, GordonMillay, Douglas P.Veenstra, Jan A.Franco, DiegoMillecamps, MagaliVeeranki, SudhakarFrandsen, Henrik LauritzMilledge, John J.Velasco Ortega, EugenioFrank, SašaMiller, Aaron W.Velez, ZéliaFranklin, GregoryMiller, Donald W.Velluto, LuciaFranklin, Michael J.Miller, IngridVeloso, Fernando T.Franklin, Rima B.Miller, JessicaVeloso, JavierFrankowski, MarcinMiller, VandanaVenier, PaolaFranssen, FritsMills, DavidVenturini, DanielleFranssen, Maurice C.R.Mills, Kenneth V.Venukadasula, PhanindraFranzke, Claus-WernerMilosevic, AnaVerardo, VitoFrauman, Albert G.Min, Jun-KiVerburg, FredericFreedman, Mark StevenMinagar, AlirezaVerderio, ClaudiaFreije, José M.P.Minami, TakashiVerderio, PaoloFreitas, Ana C.Minamino, TetsuoVerdolotti, LetiziaFrenking, GernotMinardi, DanieleVergara, AlessandroFresta, MassimoMinehan, Thomas G.Vermeulen, LouisFreund, DanaMinella, AlexVernhettes, SamanthaFreund-Levi, YvonneMinocha, Subhash C.Verron, EliseFriák, MartinMinuk, GeraldVerstraelen, ToonFribley, Andrew M.Miranda, CarlosVetvicka, VaclavFridman, GregoryMirande, MarcViana Baptista, PedroFriedmann, AntonMirzayans, RazmikViappiani, CristianoFroeyen, MatheusMitani, AkioVicente, Cláudia S.L.Fröhlich, EleonoreMitra, AbhisekVickaryous, Matthew K.Frolov, AndrejMittal, SandeepVickers, MarkFrost, SofiaMiura, YoshikoVidic, JasminaFu, Shu ManMiyamoto, HiroshiViehweger, KatrinFujii, TakaoMiyamoto, KeiVieira, Cristina P.Fujimori, KoMiyamoto, KojiVieira, Helena L.A.Fujita, MasayukiMiyamoto, TakeshiViggiano, DavideFujita, NaokoMiyaoka, HiroakiVilagran, I.Fujita, TetsujiMiyata, JunVilke, Gary M.Fujita, YuichiMiyazaki, TeruoVillacorta, LuisFujiwara, HironoriMiyoshi, DaisukeVillanneau, RichardFujiwara, RyoichiMizuguchi, MineyukiVillari, ValentinaFujiyama, KazuhitoMizuno, TetsuyaVillena, Josep A.Fukagawa, TatsuoMizuta, S.Viluksela, MattiFukoura, HirotaroMo, Yin YuanVincent, Stéphane D.Fukui, HirokoMoats, Rex A.Viola, GiampietroFukunishi, YoshifumiMoazedi-Fuerst, F.Viscomi, Maria TeresaFuller-Espie, Sheryl L.Mocanu, G.Visser, LydiaFulzele, Sadanand T.Mocchegiani, EugenioVistoli, GiulioFurneri, Pio MariaMocellin, SimoneVitali, JacquelineFuso, AndreaMockaitis, KeithanneVitali, MatteoGaerstel, MatthiasModirrousta, MandanaVitetta, LuisGaiddon, C.Modugno, FrancescaVizan, PedroGailer, JürgenMognato, MaddalenaVladimirov, Vladimir I.Gaitzsch, JensMohammad, Ramzi M.Vogel, UllaGaj, ThomasMohammadi, Alireza M.Vogl, ThomasGal, A.Mohanam, SanjeevaVolbracht, ChristianeGalanakis, CharisMohanta, TapanVon Delius, MaxGaleone, AntonioMohr, FabianVon Kalm, LaurenceGalibert, FrancisMoioli, BiancaVon Knethen, AndreasGalli, AndreaMolasiotis, AthanasiosVon Szentpály, LászlóGallo, AntonioMoldzio, RudolfVondrasek, JiriGalvão, Adelino M.Molina, Juan JesusVonk, L.A.Gálvez, JulioMolina-Bolívar, J.A.Voronov, IrinaGalvez-Valdivieso, G.Møller, Ian M.Voss, RandalGalzitskaya, Oxana V.Molvarec, AttilaVoz, Marianne L.Gamal, NesrineMolyneux, KarenWachten, DagmarGambardella, AntonioMonaco, Edward A., IIIWada, JunGambino, G.Monji, AkiraWagner, Amy K.Gammone, M.A.Monk, JenniferWagner, AnikaGams, WalterMonsees, ThomasWagner, BrentGan, WenjianMontagut, ClaraWagner, CarolGanguli-Indra, GitaliMonteil-Rivera, FannyWagner, DavidGanini, DouglasMonteiro Ramalhinho, A.C.Waheed, AbdulGanta, Chanran K.Montesinos-Pereira, D.Waite, Matthew M.Gao, LiangliangMonti, DanielaWakabayashi, Ken-ichiGao, XiaohuaMonti, Maria ChiaraWakino, ShuGao, XueMontoliu, LluisWalker, CeliaGarcía Morote, F.A.Moon, Hyung-InWalker, DavidGarcía, TaniaMoon, Il SooWalker, Lary C.García-Caballero, MelissaMoon, JisookWallace, G.Garcia-Moreno, DianaMoon, Young-AhWallace, Maeve E.Garcia-Orad, AfricaMorales, María Belén L.Wallentin, LarsGarcía-Osorio, CesarMorales, PatriciaWallhäusser-Franke, E.Garcia-Salcedo, Jose A.Moran, Corey S.Walsh, DavidGard, PaulMorandi, LucaWalther, DirkGardner, Andrew W.Morbidelli, MassimoWambach, JenniferGardner, Peter J.Moreau, RegisWan, Jian-boGarg, Parveen K.Moreira, J.B.N.Wan, xiaochunGargiulo, GaetanoMorell, ChristopheWanasundara, JanithaGarrabou, GlòriaMorello, RoyWandosell, F.Garriga, PereMoretti, Marcelo L.Wang, Bo-ChengGary-Bobo, MagaliMorgan, ClaireWang, Chin-KunGasque, PhilippeMorgan, WilliamWang, ChunlingGatti, LauraMorgante, FrancescaWang, DongGayral, PhilippeMori, HitoshiWang, FengshanGazdhar, AmiqMoriguchi, TakayaWang, HaiboGazouli, MariaMorikawa, HisaoWang, Han-ChingGe, YubinMorikis, DimitriosWang, HuizhiGeerts, HugoMorillon, R.Wang, JianGeiser, DawnMorishita, RyuichiWang, Jian-WenGeisler-Lee, JaneMorita, KyojiWang, JianyingGeissler, NicoleMörl, MarioWang, JinhuGelmann, EdwardMormile, RaffaellaWang, JunjianGenovese, GiuseppaMoro, LoredanaWang, JunjieGentile, PiergiorgioMorone, PiergiuseppeWang, KaiGeorge, Kimberly S.Morotti, AlessandroWang, P.Georgiou, IoannisMöröy, TarikWang, PengGepts, PaulMorris, Brian J.Wang, QunGer, T.R.Morris, DylanWang, RuiGeraci, FabianaMorris, JohnWang, RuoxiangGeraldes, PedroMorris, MatthewWang, Timothy C.Gerhard, MarkusMorris, May C.Wang, Tsung-ShingGerke, JörgMorrison, DouglasWang, Tzu-haoGermain, DorisMorsczeck, ChristianWang, WenxinGermershaus, OliverMort, John S.Wang, Xiang SimonGershburg, E.Mortier, JérémieWang, XiaohongGershon, TimothyMoseke, ClausWang, XinkunGeuna, StefanoMoshaverinia, AlirezaWang, XungaiGewirtz, David A.Moss, Alan C.Wang, YaGhanotakis, Demetrios FMota, ManuelWang, YadongGharbi, Y.Mott, Justin L.Wang, Yan-HsiungGhelardi, EmiliaMotterlini, RobertoWang, YingGhersi, DarioMou, Chung-YuanWang, Ying-JanGhiladi, Reza A.Moulis, Jean-MarcWang, YonghuaGhoraani, BehnazMouradov, AidynWang, YuechunGhose, RanajeetMoxon, Joseph V.Wang, Yun-MingGiaginis, ConstantinosMudaliar, SunderWang, ZhangGiampiei, FrancescaMudalige, Thilak K.Wang, ZhiguangGiannaccini, MartinaMueller-Roeber, BerndWang, Zhong Q.Giannattasio, SergioMuir, KennethWanke, DierkGiaouris, EfstathiosMuise-Helmericks, R.C.Warby, Simon C.Gibot, LaureMukaida, NaofumiWard, Peter A.Giera, MartinMukherjee, TusharmouliWarren, Gregory L.Giesbrecht, Gerald F.Mukhopadhyay, ParthaWarzecha, TomaszGilbert, NickMukudai, YoshikiWasmuth, SusanneGilch, SabineMüller, DafneWatanabe, MasakatsuGiles, Wayne R.Muller, SylvianeWatanabe, MasatoshiGill, Chris IrMüller-Buschbaum, PeterWatanabe, ToruGillings, MichaelMulner-Lorillon, OdileWatanabe, ToshioGilson, MichaelMunang’andu, Hetron MWatanabe, YuichiroGiol, Elena DianaMunoz, MacarenaWatashi, KoichiGiordano, AntonioMuñoz-Torres, ManuelWätjen, WimGiovarelli, MirellaMunshi, Nikhil V.Watson, ChristaGirao, HenriqueMuraguchi, HajimeWeathers, Palmela J.Giridhar Babu, A.Murai, KojiWebb, I.C.Girón-González, J.-A.Murakami, KazumaWeber, Franz E.Gisselmann, GüntherMurakami, YasufumiWeber, J. MarkGiuliani, DanielaMurashita, KojiWeber, JostGjini, EvisaMurata, YoshiyukiWebster, Marie R.Glauser, GaetanMuroi, CarlWeese, ScottGleich, Gerald J.Murono, ShigeyukiWegiel, BarbaraGlembotski, Christopher C.Murray, AjaWei, Guor-JienGlocker, Erik-OliverMurray, Brent W.Wei, JingGloria, AntonioMurray, Christopher JWeigand, AnnikaGnana-Prakasam, JayaMurray, James D.Weigand, Julia E.Goberdhan, Deborah CIMurray, MeganWeiler, HartmuGobinet, CyrilMurray, PatriciaWeiss, JuliaGodin, Bruno GodinMurrell, Dédée F.Weiss, NorbertGoergen, Craig J.Murtas, DanielaWeitzel, Joachim M.Goglia, FernandoMurugaiyan, JayaseelanWen, C.M.Gojo, S.Muschel, Ruth J.Wen, ZhiyouGoldberg, Inna KhozinMusumeci, GiuseppeWeng, Ching-FengGolding, Jon P.Musumeci, TeresaWenger, Nanette K.Goldman, Myla D.Muthusamy, Visaliniwenlong, ChangGoldoni, MatteoMuto, TomoyukiWerling, DirkGoldring, MaryMutoh, TatsuroWerner, Rudolf A.Goldstein, Aaron S.Muylaert, KoenraadWertz, PhilipGoldstein, Benjamin I.Muyldermans, SergeWestendorf, JohannesGoldup, StephenMuzzarelli, Riccardo A.A.Wetterberg, LennartGolestaneh, NadyMyklebost, OlaWheeler, ThomasGollasch, StephanMyung, Pyung-KeunWhisner, CorrieGolledge, JonathanNadal, PolWhite, JasonGoltsov, AlexeyNaegele, R.P.Whiteside, Theresa L.Goltz, DianeNag, Dilip K.Whitford, Paul C.Gomer, Richard H.Nagai, KouheiWiborg, OveGomes, Helder T.Nagano-Takebe, FutamiWidhalm, GeorgGómez, GustavoNagasawa, HiromichiWidhalm, KurtGómez-Jeria, J.-S.Nagata, TakeshiWiechec, EmiliaGómez-Laguna, J.Nagatomo, ShigenoriWiegertjes, GeertGonçalves, Ana LuísaNagy, IstvanWielstra, BenGonçalves, António PedroNahashon, SamuelWildemann, BrittGonçalves, OlivierNair, AshwinWilharm, GottfriedGondhalekar, Ameya D.Nair, Lakshmi S.Wilkinson, J.M.Gondi, Christopher S.Naito, KenWilliams, David C., Jr.Góngora-Castillo, ElsaNaito, YujiWilliams, Thomas L.Gonzales, EricNajimi, MustaphaWilson, Adam C.Gonzalez Muniesa, PedroNaka, HiroakiWilson, D.B.González, María PilarNaka, TetsujiWilson, Karl A.Gonzalez, RamonNakagawa, YusukeWilson, MichaelGonzález, Víctor M.Nakai, MasaakiWilson, RobertGonzález-Bello, C.Nakaki, ToshioWiltshire, Esko J.González-Díaz, H.Nakamoto, ShingoWimmers, K.González-Maeso, JavierNAKAMURA, KazuhikoWingert, Rebecca A.Goossens, N.Nakamura, KazukiWinkler, G. SebastiaanGordon, Catherine A.Nakamura, KozoWinkler, ThomasGorgoulis, Vassilis G.Nakamura, NorikoWinship, Ian R.Gori, TommasoNakao, MinoruWinssinger, NicolasGorinstein, ShelaNakashima, KazuoWinterbone, MarkGorman, ShelleyNakatani, YoshihikoWirth, D.Goswami, A.Nakayama, KohWischhusen, JoergGothilf, YoavNakayama, YasumuneWitt-Enderby, PaulaGoto, KazuoNakayama, YujiWitzel, KatjaGoto-Inoue, NaokoNamihira, MasakazuWoeltje, MichaelGotor-Fernández, VicenteNanashima, AtsushiWoisel, PatriceGott, Ryan C.Nånberg, EewaWold, William S.M.Gottesman, Michael M.Nandrot, Emeline F.Wolf, Jennifer MoriatisGoulão, Luís Filipe S.Narayana, AshwathaWolfl, C.Gould, GwynNarod, StevenWolfson, AdiGourrierec, José LeNasrallah, June B.Woloszyk, AnnaGovindarajulu, R.Nasri, RimWon, Chong HyunGowans, GordonNatarajan, Sathish KumarWong, Albert H.C.Grabrucker, AndreasNatesan, SenthilWong, Anderson On LamGraca, LuisNaughton, PatrickWong, Boon-SengGracias, DavidNaumann, Todd A.Wong, Chi-MingGrady, Bart P.X.Naumova, Anna V.Wong, K.-K.Graether, Steffen P.Navarra, MicheleWong, Kenneth K.Y.Graham, AnnNavarro, MiguelWong, MelanieGraham, NeilNaviglio, SilvioWong, Vincent Kam WaiGrammatikakis, IoannisNaya, Francisco J.Woo, Hee ChulGramzow, LydiaNazar, Ross N.Woo, So-YounGranata, SimonaNeacsu, MadalinaWood, W. GibsonGraner, MichaelNeerinckx, BarbaraWoodfield, TimGranitzer, PetraNegi, Surendra S.Woodfield, Tim B.F.Grant, WiliamNegro, EnricoWoods, MatthewGrassi, GabrieleNeier, ReinhardWoody, Robert W.Grattagliano, IgnazioNeirinckx, VirginieWorth, AndrewGray, Neil D.Nellaiappan, K.Wright, LouwranceGraziano, GiuseppeNemunaitis, JohnWu, Cheng-HsunGraziano, GuellaNesmelov, Yuri E.Wu, Chia-WenGrebien, FlorianNeugart, SusanneWu, Huey-NanGreen, CharlotteNeuhauss, StephanWu, Jian-PingGreenberger, Joel S.Neutzner, AlbertWu, Kenneth K.Greene, Lesley H.Neuzil, JiriWu, KeqiangGreenidge, P.A.Nguyen, PhuongWu, LianfengGreening, David W.Ni, WeimingWu, Ming-jiuanGreenwood, DavidNicassio, FrancescoWu, MoushengGreenwood, Michael T.Nichols, A.R.L.Wu, NicholasGreim, HelmutNicholson, Allen W.Wu, PenseeGresshoff, PeterNicolás, Francisco E.Wu, Ren-JangGrey, CarlNiculescu, Mihai D.Wu, Sheng-ChiGrieco, Carmine R.Niel, Ed vanWu, Shih-HsiungGrieco, FrancescoNiemann, BerndWu, Shu-YiiGriese, MatthiasNieminen, Anna-LiisaWu, Tzong-YuanGrignani, G.Nigatu, Yeshambel T.Wu, XiaoqingGrimholt, UnniNiidome, TakuroWu, Y.Grimley, Philip M.Niimi, TomoakiWu, YuliangGrimpe, BarbaraNiino, MasaakiWung, Being-SunGrinberg, DanielNijnik, AnastasiaWursthorn, KarstenGrinfeld, JacobNikamo, PernillaWürtz, MortenGris, Jean ChristopheNikolakakis, IoannisWysocki, Lawrence J.Grisoni, FrancescaNikolic, DejanWyss, ChristineGroenendyk, JodyNilsson, PerXiao, J.Gromak, NataliaNinfali, PaolinoXiao, LiGros, FrédéricNing, BaitangXiao, ZhoushengGrosso, ClaraNiranjan, RiturajXie, GuofengGrosso, GiuseppeNishida, YoshihiroXodo, Luigi E.Gruh, InaNishihara, TatsujiXu, BaoshanGrusch, M.Nishina, HiroshiXu, LeyuanGsponer, JörgNishio, ZentaXuan, XiangchunGu, Hong-chenNishiwaki, HisashiXuan, ZhenyuGu, JianNishiyama, ToshioXue, BinGu, YanNisnevitch, MarinaXue, MeilangGuaricci, Andrea IgorenNissilä, Marika E.Xue, XiangGudiña, EduardoNistala, RaviYada, TakashiGueimonde, MiguelNkeng-Efouet, Pepin A.Yagi, HiroshiGuénal, I.Nobili, StefaniaYahubyan, GalinaGuentsch, ArndtNoguera, Daniel R.Yamabe, ShinichiGuerra, DavideNoh, HyunjinYamada, SohsukeGuerre-Millo, MichèleNojiri, TakashiYamagishi, AkihikoGuerzoni, Maria E.Nomoto, ShujiYamaguchi, HiroyukiGuglielmi, P.O.Nomura, M.Yamaki, KojiGuillaume, SophieNonami, AtsushiYamamoto, AkihiroGuilliams, M.Noordzij, WalterYamamoto, MasayukiGuillot, BertrandNorman, PeterYamamoto, NorioGuin, SunnyNorman, RobertYamamura, SoichiroGulbinsh, ErichNormand, PhilippeYamano, KojiGulino, RosarioNorrild, BodilYamauchi, SatoshiGunaje, JayaramaNorval, MaryYamauchi, TakahiroGunkel, GünterNothnick, Warren B.Yamaza, TakayoshiGuo, LinNouri, KeyvanYan, BowenGuo, Li-TaoNouri, M.-Z.Yan, JunGuo, MinNovak, JohannesYan, Liang JunGuo, PeixuanNowikovsky, KarinYan, ShanGuo, XingNuevo, MichelYang, Da-QingGuo, Z. ShengNumakawa, TadahiroYang, Eddy S.Gupta, AnkitNunney, Leonard N.Yang, FanGupta, SanjayNunzio, Mattia DiYang, Guang-dongGupta, SudhiranjanNussler, Andreas K.Yang, HongshunGupta, Vivek K.Nylander, KarinYang, Jen-ChangGurevich, Eugenia V.Nyström, Andreas M.Yang, MinoYangGurevich, Vsevolod V.Nyström, KristinaYang, PingfangGursky, OlgaO’Carroll, Simon J.Yang, Seong WookGurzov, Esteban N.O’Donnell, Kieran J.Yang, Wei-hsiungGutekunst, KirstinOakeson, Kelly F.Yang, Weng-LangGuthridge, Mark A.Oakley, Brian B.Yang, XiaoyanGutiérrez Martín, S.Oancea, ElenaYang, YongGutschner, TonyO'banion, MichaelYang, YongguangGuttery, DavidObare, SherineYang, YuedongGyselaers, W.Obdeijn, M.C.Yang, YuzheHaapalainen, MinnaOberholster, AnitaYano, ShozoHaapasalo, AnnakaisaOchiya, TakahiroYao, IzumiHaas, HubertusOcio, EnriqueYao, WeiHaase, HajoOetting, William S.Yao, Zhong-PingHaass, Nikolas K.Ogata, TadanoriYasuda, KazukiHackenberg, MichaelOgata, YorimasaYasuhara, TakaoHadjiargyrou, MichaelOgawa, NorikoYasuko, ManabeHadjipavlou-Litina, D.Ogawa, RyuichiYazlovitskaya, E.M.Hadwiger, LeeOgawa, TomohisaYeddula, NarayanaHadziyannis, EmiliaOgawa, Y.Yeh, Chau–TingHaenen, Guido R.M.M.Ogawa, YukoYeh, Jui-MingHagedoorn, Peter-LeonOgi, KazuhiroYeh, Jwu-LaiHagenacker, TimOgilvie, CarolineYeh, Kuo-ChenHahn, MatthiasOgino, ShujiYeh, Min-LongHahn, Sei KwangO'Gorman, David B.Yellon, Derek M.Hai, BoOgra, YasumitsuYen, Feng-LinHaider, NorbertOgunjirin, Adebowale E.Yennu-Nanda, V.G.Hajnsdorf, ElianeOh, Jae-MinYeoman, CaarlHaldrup, K.Oh, Sae-ockYeung, Kam C.Halford, Nigel G.O'Hara, LauraYeung, Sai-Ching JimHalina, Machelska-SteinOhi, KazutakaYezierski, Robert P.Hall, Joseph C.Ohkohchi, NobuhiroYilmazel, Yasemin D.Hallegraeff, Gustaaf M.Ohlendieck, KayYin, JohnHallouard, FrançoisOhnishi, MasatoshiYing, Shao-YaoHalonen, Sandra K.Ohnuma, TohruYokobori, TakehikoHamamura, K.Ohta, TakeshiYokoi, TsuyoshiHamann, MartineOhtaki, HirokazuYokota, ShinsoHamdy Roby, Mohamed H.Okabayashi, JunYokoyama, TomoyukiHamerlynck, Erik P.Okawa, MasakazuYokoyama, YoshihitoHammond, JohnOkazaki, ToshiroYonekura, ShinichiHamrah, P.OKUMURA, NobuakiYoneyama, TadakatsuHamzeh, MahsaOkuyama, HidetoshiYoo, DongwanHan, Dong WookOliveira, AndreiaYoo, Eun-SookHan, Inn-ocOliveira, P.A.Yoo, Wan-HeeHan, JinOliviero, F.Yoon, Do-YoungHan, QuanbinOllas, Carlos deYoon, Hwan SuHan, Sang-baeOllikainen, MiinaYoon, JaeseungHan, Seung HyunOlson, Michael F.Yoon, Je-HyunHan, Yeon SooOlson, Peter D.Yoon, MinjoongHanagasi, Hasmet A.Olsson, ChristianYoon, Moon-YoungHanaoka, MitsumasaOltra, ElisaYoon, Seog-youngHanawa, HaruoOmetto, F.Yoon, Shin H.Hanazono, YutakaOndruschka, BenjaminYoshida, HiderouHanhineva, KatiOnozaki, KikuoYoshida, HiroshiHann, Hie-WonOono, YoukoYoshida, KunihiroHannafon, BethanyOostenbrink, ChrisYoshii, ToshitakaHannan, Anthony J.Oosterkamp, MargreetYoshikawa, MasaakiHannestad, JonasOosterwijk, EgbertYoshimitsu, TakehikoHanschen, Franziska S.Ordi-Ros, JosepYoshimura, AkihikoHansen, T.F.O'Reilly, Michael A.Yoshimura, KazuyaHarada, H.Oresnik, Ivan J.Yoshimura, KoichiHarada, MamoruOrlova, Elena V.Yoshioka, HideyukiHarada, NaokiOrner, Brendan P.Yoshitomi, HiroyukiHarada, YoshieOrtiz, Gabriela ToledoYou, LiangHaraguchi, YujiOrtiz, InmaculadaYoung, Robert S.Haraldsen, GuttormOrtiz-Soto, MariaYoza, Brandon A.Harari, DanielOrtlund, Eric A.Yperman, JanHarauz, GeorgeOsada, JesúsYu, Cheng-ChiaHarb, JamilOsanai, T.Yu, Cheng-PingHardingham, Jennifer E.Oshitari, ToshiyukiYu, Chia-LiHardtke-Wolenski, M.Oster, HenrikYu, Chin-shengHardy, John GeorgeO'Sullivan, CathrynYu, FengqunHardy, MargaretOsumi, KenjiYu, Jae-RanHare, Dominic J.Ota, MotonoriYu, Jennifer S.Hare, Joshua M.Otsuka, FumioYu, LijianHarn, Chee HarkOttinger, Elizabeth A.Yu, MengxiaoHarris, Jennifer W.Oudit, Gavin Y.Yu, Yan PingHarris, LindaOuzounidou, GeorgiaYu, YongjunHarrison, Marcia A.Owens, LeighYu, Yongxin JeremyHarrison, Paul H.M.Oyama, FumitakaYuan, XiaoHarrison, RogerOzaki, IwataYuasa, S.Hart, David A.Ozaki, ToshinoriYue, PatrickYingKitHart, RonaldOzawa, KoichiroYuen, TonyHartnett, David C.Ozeki, YasuhiroYuh, Chiou-HwaHartz, Kara E. HuffOzeki, YoshihiroYull, Fiona E.Haruki, MitsuruPace, VittorioYun, DaiHarvey, RichardPadhye, NikhilYun, Hae KeunHasanuzzaman, MirzaPadilla, DanielYun, Jong WonHasegawa, TomonobuPadmalayam, IndiraYunus, AghaHasegawa, YasushiPadmanabhan, JayaYusupova, GulnaraHaspel, NuritPadula, MatthewZabel, Brian A.Hassan, MohamedPaeshuyse, JanZabetakis, IoannisHassel, BretPagan, OneZablackis, EarlHatae, NoriyukiPaik, JisunZaccone, ClaudioHatakeyama, KatsunoriPal, RiturajZachara, Natasha E.Hati, SanchitaPalazzetti, FedericoZachut, MayaHatle, J.Palazzo, Alexander F.Zago, MiriamHauber, JoachimPalenzuela López, José A.Zagouri, FloraHaughton, DavidPallauf, KathrinZahid, MalihaHavird, Justin C.Pallavicini, AlbertoZakeri, BijanHayakawa, TohruPalm, Gottfried J.Zakhartchouk, AlexanderHayes, Don, Jr.Palm, ThomasZaknun, John J.Hayes, MariaPalumbo, CarlaZalapa, Juan ErnestoHayes, RichardPan, Chun-HsuZamaratskaia, GaliaHaynes, Cole M.Pan, Min-HsiungZan, JindongHazlett, Linda D.Panaro, Maria AntoniettaZanke, BrentHe, HongPanda, Amaresh C.Zannad, FaiezHe, Quan SophiaPañeda, CovadongaZannas, Anthony S.He, SongbingPanfoli, IsabellaZannoni, Gian FrancoHeadon, Denis J.Pang, YiZanotti, GiuseppeHearing, PatrickPanico, AntonioZaravinos, ApostolosHeath, P.R.Pantano, PatriziaZarbock, RalfHebbard, L.Pantos, ConstantinosZarogoulidis, PaulHedhammar, MyPantoș, G. DanŽárský, ViktorHedley, PeterPanzella, LuciaZavitsas, AndreasHeegaard, Niels H.H.Paolocci, NazarenoZehbe, RolfHefferon, Kathleen LauraPapaccio, GianPaoloZeilinger, CarstenHelariutta, YrjöPapagerakis, PetrosZelarayán, Laura C.Helder, MarcoPapait, RobertoZenarruzabeitia, OlatzHeldring, NinaPapassiopi, NymphodoraZeng, HuaqiangHellweg, Christine E.Papathanos, PhilipposZeng, LingfangHenao-Martinez, Andres F.Papi, AlessioZeng, Xin-XinHendricks, TheoParadisi, FrancescaZerbini, GianpaoloHendrickson, C.L.Parahuleva, Mariana S.Zervos, Antonis S.Hendriksen, Peter J.m.Pardo, MariaZgaga, LinaHennebelle, ThierryParenti, Serena IncertiZgaljardic, Dennis J.Hennies, Hans ChristianParini, RossellaZhang, CaiguoHennig, FraukePark, Chail-IlZhang, CanHenrich, BirgitPark, EnochZhang, ChengfeiHenrich, Curtis J.Park, EugeneZhang, HaoqianHenry, MorganePark, Hun-kukZhang, QiHera, ConchaPark, In-KyuZhang, QingHerath, TharanganiPark, Jeong-sookZhang, ShaopingHerbert, Karl HerbertPark, Jong-ChulZhang, TuoHeredia, Unai LópezPark, Jong-HoonZhang, XuchenHerington, Jennifer L.Park, Ky YoungZhang, XuehongHermans, EmmanuelPark, SunghyoukZhang, XunHernández, J.A.Park, Tae JungZhang, YanjinHernandez, PilarPark, Woo HyunZhang, YongHernandez-Ledesma, B.Park, YongsoonZhang, ZhenhuaHerndon, DavidParker, Anthony W.Zhao, LiangHerranz, Raul CanoParks, RobinZhao, Shou-JingHerrmann, AndreasParnell, Jill A.Zhao, YoufuHerrmann, MartinParniakov, OleksiiZhbannikov, Ilya Y.Herse, FlorianParodis, IoannisZheng, GuopingHerst, PatriesParrella, PaolaZheng, WenguangHerzog, FabianParrish, AlanZhong, XueHesser, HugoParsonage, DerekZhou, BeiyanHettich, RobertParsons, Barry J.Zhou, BingHeuberger, AdamPasán, JorgeZhou, Binhua P.Heyd, FlorianPascault, Jean-pierreZhou, DaohongHeymans, StephanePascual-Teresa, SoniaZhou, HelingHeyninck, KarenPasini, DarioZhou, HuanHiblot, JulienPasini, LuigiZhou, ManHiggins, Paul J.Pask, Andrew J.Zhou, QingxiangHighley, John RobinPassi, AlbertoZhou, WeijunHiguchi, AkonPastor, IsabelZhou, WenguangHili, RyanPastor, JesúsZhou, XiangrongHill, Jeff W.Patarrão, RitaZhu, ChangfuHiltermann, T. Jeroen N.Pater, Sylvia deZhu, Meng-YangHimeno, SeichiroPathak, HarshZhu, Qian-HaoHinds, Terry D., Jr.Patil, AshwiniZhu, XiaoshanHinkel, RabeaPatriarca, CarloZhu, YefeiHinsen, KonradPattenden, GeraldZhuang, JieHinton, CimonaPatti, FrancescoZiai, Wendy C.Hirahara, KiyoshiPattison, David I.Zignego, Anna LindaHirasawa, NoriyasuPaulmurugan, RamasamyZinzen, RobertHirashima, AkinoriPaulsen, BeritZiparo, ElioHirayama, FumitoshiPaulson, Elizabeth L.Zivadinov, RobertHiroaki, FujiiPavel, Ioana E.Zodrow, Erwin L.Hirsch, Anna K.H.Pavert, Steven H. P. van deZolotukhin, SergeiHirsch, Rhoda ElisonPavlov, Youri I.Zorzano, AntonioHisabori, ToruPavon-Djavid, GracielaZou, ShengliHjalmarsson, ClaraPavone, Luigi MicheleZou, XueqingHo, Michael (Shan-Hui)Pawlak, RémyZsurka, GaborHo, VikkiPazzola, MicheleZucman-Rossi, JessicaHo, Wen-FuPearson, James T.Zullo, LetiziaHocher, ValeriePedeux, RémyZuo, YuegangHodgson, LouisPeijnenburg, Willie J.G.M.Zuppinger, ChristianHodson, LeannePeker, DenizZwart, Mark P.Hoemann, Caroline D.Pekkinen, MinnaZwaveling, J.Hoffjan, Sabine



